# Knocking at the Doors of Perception: Relating LSD Effects on Low‐Frequency Fluctuations and Regional Homogeneity to Receptor Densities in fMRIf

**DOI:** 10.1111/ejn.70338

**Published:** 2025-11-26

**Authors:** Paolo La‐Torraca‐Vittori, Livio Tarchi, Elisa Arrigo, Stefano Lanterna, Eleonora Tosi, Arne Doose, Fulvia Palesi, Doris Pischedda, Valdo Ricca, Paolo Fusar‐Poli, Stefano Damiani

**Affiliations:** ^1^ Department of Brain and Behavioural Sciences University of Pavia Pavia Italy; ^2^ Psychiatry Unit, Department of Health Sciences University of Florence Florence Italy; ^3^ CIMeC—Center for Mind/Brain Sciences University of Trento Rovereto Italy; ^4^ Division of Psychological and Social Medicine and Developmental Neurosciences, Translational Developmental Neuroscience Section, Faculty of Medicine Technische Universität Dresden Dresden Germany; ^5^ Department of Psychosis Studies King's College London London UK; ^6^ Outreach and Support in South‐London (OASIS) Service, South London and Maudlsey (SLaM) NHS Foundation Trust UK; ^7^ Department of Psychiatry and Psychotherapy Ludwig‐Maximilian‐University Munich Germany

**Keywords:** ALFF, consciousness, lysergic acid diethylamide, neuroimaging, psychedelics, ReHo

## Abstract

Despite a renewed scientific interest in lysergic acid diethylamide (LSD), its local neural effects remain underexplored. This functional magnetic resonance imaging (fMRI) study explored and compared LSD‐induced changes in local activity (amplitude of low‐frequency fluctuations: ALFF) and local connectivity (regional homogeneity: ReHo), assessing their relationship to regional receptor density. Imaging data of 15 healthy adults from an open dataset were analyzed. For each participant, two pairs of resting‐state runs were available (rest1 and rest2), one performed under placebo and one following the intravenous administration of 75‐μg LSD. Voxel‐wise paired *t*‐tests compared ALFF and ReHo in the LSD versus placebo conditions. Rest1*rest2 test–retest reliability and ALFF*ReHo cross‐modal associations were assessed with conjunction maps and vertex‐wise correlations. Finally, neurochemical enrichment analyses related LSD‐induced ALFF and ReHo changes to cortical density maps of LSD‐related neurotransmitter receptors and transporters. Both ALFF and ReHo decreased in somatosensory/visual cortices under LSD compared to placebo. Specific decreases were observed for ALFF in associative regions belonging to the default mode and frontoparietal networks, and for ReHo in subcortical regions (cluster‐based corrected *p* < 0.05). Test–retest reliability was high for ALFF (rho = 0.80, *p* = 0.001) and moderate for ReHo (rho = 0.46, *p* = 0.001). ALFF*ReHo LSD‐induced changes were moderately associated (rest1: rho = 0.36, *p* = 0.001; rest2: rho = 0.56, *p* = 0.001). Neurochemical enrichment analysis showed that LSD‐induced ALFF/ReHo alterations were reliably and negatively correlated with the density of D2 and 5‐HT1A receptors (FDR‐corrected *p* < 0.05). These preliminary findings suggest that LSD may engage complex and dynamic neurochemical processes beyond its known 5‐HT2A receptor target, warranting further investigation.

## Graphical abstract

LSD induced widespread and reliable reductions in ALFF and ReHo. ALFF decreases mainly involved associative cortices, while ReHo reductions predominated in sensory and subcortical regions. These neural alterations negatively aligned with D2 and 5‐HT1A receptor distributions, suggesting that LSD may engage complex and dynamic neurochemical processes beyond its known 5‐HT2A receptor target, warranting further investigation.

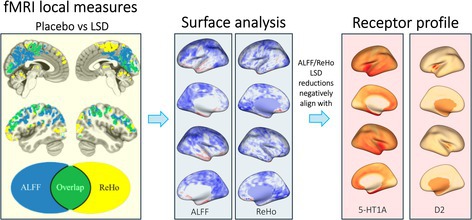

**Abbreviations**
5‐HT1/2/4: 5‐Hydroxytryptamine (Serotonin) receptors (type 1,2,4)
ALFF: amplitude of low‐frequency fluctuations
BOLD: blood oxygen level dependent (signal)
D1, D2: dopamine receptors (type 1 and 2)
dlPFC: dorsolateral prefrontal cortex
EBH: entropic brain hypothesis
fALFF: fractional ALFF
FC: functional connectivity
fMRI: functional magnetic resonance imaging
IFG: inferior frontal gyrus
LSD: lysergic acid diethylamide
mALFF: mean‐normalized amplitude of low‐frequency fluctuations
PBO: placebo
PPC: posterior parietal cortex
ReHo: regional homogeneity
SMA: supplementary motor area
SMC: sensorimotor cortex

## 1 Introduction

Lysergic acid diethylamide (LSD) is a compound classified among serotonergic, or “classic,” psychedelics—a category that includes, among others, psilocybin, ayahuasca, and mescaline. Its effects are currently thought to be primarily mediated by agonist activity at the serotonin 5‐HT2A receptor (Dai et al. 2023; Shinozuka et al. 2024). Since its discovery in the 1950s, LSD has been one of the first psychedelics to demonstrate potential therapeutic benefits (Grinspoon and Bakalar 1997; Kyzar et al. 2017; Linguiti et al. 2023). However, research on LSD has historically been constrained by strict regulatory barriers, compounded by its widespread recreational use and abuse (Kyzar et al. 2017). Recently, there has been renewed scientific interest in LSD and its neural correlates, driven by its low addictive potential combined with promising efficacy in the treatment of various psychiatric disorders (Linguiti et al. 2023; McCulloch et al. 2022).

Previous fMRI studies have consistently demonstrated a global pattern of reduced within‐network functional connectivity (FC) (often referred to as “integrity”), decreased segregation (or “modularity”), and increased between‐network FC during the acute administration of LSD (Bedford et al. 2023; Carhart‐Harris et al. 2016; Dai et al. 2023; Linguiti et al. 2023; Luppi et al. 2021; McCulloch et al. 2022; Moujaes et al. 2024; Müller et al. 2018; Tagliazucchi et al. 2016).

In parallel with FC studies, the recent formulation of the entropic brain hypothesis (EBH) has provided another important framework for understanding the effects of psychedelics on brain dynamics (Carhart‐Harris et al. 2014; Carhart‐Harris 2018). In formal terms, entropy is a scientific concept commonly associated with states of disorder, randomness, or uncertainty. In neuroscience, entropy‐based metrics such as Shannon entropy—measuring the uncertainty or diversity of a system's possible states (Shannon 1948)—have been applied to neural activity patterns in electrophysiological and fMRI data, with higher entropy indicating a richer, less predictable repertoire of brain states (Lebedev et al. 2016; McCulloch et al. 2022). EBH posits that brain activity during normal waking consciousness operates near a critical zone between order (low entropy) and disorder (high entropy). Several studies have reported increased brain entropy under LSD using different methodologies (McCulloch et al. 2022). Increased entropy supports a shift toward more disordered brain activity, resulting in less constrained and more responsive neural processing, as well as greater conscious content and dynamism (Carhart‐Harris 2018; McCulloch et al. 2022). However, despite significant progress in understanding LSD‐induced brain functional alterations, our knowledge remains limited by several factors.

Firstly, in contrast to the abundance of studies on long‐range FC and entropy, no research has investigated the neural correlates of psychedelics using local functional measures such as the amplitude of low‐frequency fluctuations (ALFF) (Yang et al. 2007; Zang et al. 2007) and regional homogeneity (ReHo) (Zang et al. 2004). ALFF indexes the amplitude of spontaneous neural activity. As the current theories on psychedelics hypothesize increases in neural entropy and flexibility when these substances are used, examining ALFF may offer an integrative perspective on how LSD modulates the stability of local neural dynamics. No previous study has specifically analyzed LSD‐induced ALFF alterations. However, a recent study by Delli Pizzi et al. (2023) found increased fractional ALFF (fALFF) in regions of the default mode and attention networks and decreased fALFF in limbic areas, with these spatial patterns aligning with the cortical distribution of 5‐HT2A and 5‐HT1A receptors, respectively. Although related, fALFF and ALFF can yield different results and should not be considered interchangeable (Lv et al. 2024; Merola et al. 2024; Yang et al. 2018). While fALFF measures the relative contribution of low‐frequency amplitudes to the total amplitude spectrum of the BOLD signal, ALFF provides an absolute measure of oscillatory amplitude and shows superior test–retest reliability (Jia et al. 2020; Zou et al. 2008; Zuo and Xing 2014). Regarding ReHo, previous work has suggested that reductions in local coherence may accompany decreased within‐network connectivity and increased between‐network connectivity during task state (Damiani et al. 2022; Tommasin et al. 2018). Investigating ReHo in the context of LSD is therefore of particular interest to test whether similar mechanisms—namely, a disruption of local synchrony coupled with increased global integration—extend to the psychedelic state.

Secondly, the receptor profile of LSD extends far beyond its serotonergic activity at 5‐HT2A receptors, which it shares with many psychedelics (Halberstadt and Geyer 2011; Ray 2010). In fact, LSD exhibits high affinity (Ki < 0.1 μM) for serotonergic (5‐HT1A, 5‐HT2A, and 5‐HT2C), adrenergic (ɑ2A), and dopaminergic (D2 and D3) receptors in rats (Herian 2022; Moliner et al. 2023; Rickli et al. 2015; Sonda et al. 2025). Beyond these primary targets, LSD modulates additional neurotransmitter systems. For instance, it enhances glutamatergic transmission, particularly in the prefrontal cortex (De Gregorio et al. 2016; Herian 2022; Moreno et al. 2011). Evidence for direct action at GABAergic receptors is less robust, and current consensus suggests that observed effects are circuit‐level consequences of serotonergic modulation rather than primary receptor binding (De Gregorio et al. 2016; Herian 2022). Although serotonergic stimulation may promote endogenous opioid release, LSD itself does not directly bind μ‐ or κ‐opioid receptors at relevant affinity, and opioid‐related effects are likely indirect or peripheral (Diniz et al. 2018). LSD may also indirectly inhibit acetylcholine release (Gillet et al. 1985; Jackson et al. 1988). More recently, LSD has been shown to modulate the brain endocannabinoid system, further suggesting a broad receptor profile as possibly underlying its effects (Inserra et al. 2023). In fact, notwithstanding the importance of 5‐HT2A receptors, complex interactions with other receptors may still influence the psychotomimetic effects of psychedelic drugs (Kwan et al. 2022). Apart from preliminary investigations on 5‐HT2A and 5‐HT1A (Delli Pizzi et al. 2023), no study has related LSD's functional fingerprint in fMRI to the full spectrum of its target receptors.

To address these gaps, the primary aim of this study was to compare LSD‐induced changes in local activity (ALFF) and connectivity (ReHo). ALFF and ReHo were measured in two resting‐state runs to explore the stability of the observed changes using conjunction maps between the two runs. Conjunction maps of ALFF and ReHo were analyzed to identify the regions where both measures were affected by LSD‐induced changes. As a secondary aim, neurochemical enrichment was applied to the ALFF/ReHo findings to investigate whether these measures were influenced by LSD in relation to the density of specific receptors.

## 2 Material and Methods

### 2.1 Study Participants

This study analyzed imaging data from the OpenNeuro Dataset ds003059 (Carhart‐Harris et al. 2020; Carhart‐Harris et al. 2016). The original sample included 20 subjects.

Once recruited, participants provided written informed consent and were screened for both physical and mental health conditions (Carhart‐Harris et al. 2016). Exclusion criteria included being under 21 years of age, a personal diagnosis of psychiatric illness, a family history of psychotic disorders, lack of prior exposure to classic psychedelics (e.g., LSD, mescaline, psilocybin/magic mushrooms, or DMT/ayahuasca), use of psychedelics within 6 weeks prior to the first scan, pregnancy, excessive alcohol consumption (more than 40 units per week), or any medical condition that would disqualify them from participating.

### 2.2 Informed Consent and Ethical Approval

The original study received approval from the National Research Ethics Service Committee London‐West London and was conducted in accordance with the revised Declaration of Helsinki (2000), the International Council for Harmonisation's Good Clinical Practice guidelines, and the National Health Service Research Governance Framework. The research was sponsored by Imperial College London and performed under a Home Office license for work involving Schedule 1 drugs (Carhart‐Harris et al. 2016).

### 2.3 Study Setting

Participants deemed eligible for the study attended two separate fMRI sessions, spaced at least 14 days apart. On one day, they received a placebo (PBO), and on the other, they were administered LSD. The order of these conditions was counterbalanced among participants, who remained blind to the treatment sequence, though the researchers were aware of it. On the LSD imaging days, participants were given 75 μg of LSD intravenously, delivered via a 10‐mL solution infused over a 2‐min period, followed by a saline infusion. This dosage is considered a moderate, fully hallucinogenic dose, capable of producing marked alterations in the state of consciousness (Carhart‐Harris et al. 2016; Passie et al. 2008). Participants reported experiencing the initial effects of the drug between 5 and 15 min after dosing, with peak effects occurring around 60‐ to 90‐min postdosing. MRI scanning began approximately 70‐min postdosing and lasted roughly 1 h. More information is available in the original study (Carhart‐Harris et al. 2016).

### 2.4 fMRI

Participants underwent three consecutive fMRI scans of 7:20 min each during each session: the first and third scans were resting‐state scans conducted with eyes closed, while the second scan was acquired while participants listened to music. For this study, only the resting‐state scans (no music) were included in the analysis (rest1 and rest2). Therefore, four scans were available for each subject: PBO‐rest1, PBO‐rest2, LSD‐rest1, and LSD‐rest2.

Imaging data available on OpenNeuro Dataset ds003059 were already preprocessed (Carhart‐Harris et al. 2020; Carhart‐Harris et al. 2016). More information about anatomical and data acquisition, preprocessing and motion correction procedures is reported in the Supporting Information (Sections e1–e3) and in the original paper (Carhart‐Harris et al. 2020; Carhart‐Harris et al. 2016).

### 2.5 Primary Analysis

#### 2.5.1 ALFF and ReHo

ALFF analysis (Yang et al. 2007; Zang et al. 2007) was performed using AFNI. The time series for each voxel was transformed to the frequency domain to obtain the power spectrum. Since the power of a given frequency is proportional to the square of the amplitude of this frequency component, the square root was calculated at each frequency of the power spectrum, and the averaged square root was obtained across 0.01–0.08 Hz at each voxel. This averaged square root was taken as the ALFF (Zang et al. 2007). ALFF was computed for the four runs (ALFF‐PBO‐rest1, ALFF‐PBO‐rest2, ALFF‐LSD‐rest1, and ALFF‐LSD‐rest2). As ALFF preserves the absolute amplitude of low‐frequency oscillations, making it sensitive to potential global effects of LSD, it was chosen as the primary measure. In contrast, mean‐normalized ALFF (mALFF)—defined as the ALFF of each voxel divided by the whole‐brain mean (Jia et al. 2020)—removes absolute scaling and retains only relative regional differences. To account for this complementary perspective, we also computed mALFF for all four runs (mALFF‐PBO‐rest1, mALFF‐PBO‐rest2, mALFF‐LSD‐rest1, and mALFF‐LSD‐rest2).

ReHo analysis (Zang et al. 2004) was also performed using AFNI. ReHo was calculated using Kendall's coefficient of concordance for the four runs (ReHo‐PBO‐rest1, ReHo‐PBO‐rest2, ReHo‐LSD‐rest1, and ReHo‐LSD‐rest2). Higher ReHo values reflect greater local homogeneity (Zang et al. 2004), signifying stronger correlations in the time series of a voxel with its nearest 26 neighboring voxels (Taylor and Saad 2013).

Voxel‐wise differences between ALFF‐LSD‐rest1 and ALFF‐PBO‐rest1 and ReHo‐LSD‐rest1 and ReHo‐PBO‐rest1 were assessed using paired *t*‐tests (AFNI program *3dttest++*), yielding, respectively, ALFF‐shift‐rest1 and ReHo‐shift‐rest1 (computed as LSD minus PBO). Results were then masked using a gray matter mask derived from the chosen MNI template. The same procedure was repeated for the rest2 runs, yielding ALFF‐shift‐rest2 and ReHo‐shift‐rest2. As a sensitivity analysis, we also computed mALFF‐shift‐rest1 and 2.

#### 2.5.2 Statistical Thresholding and Post Hoc Power Analysis

A cluster‐based correction was implemented using the ClustSim option in *3dttest++*, which simulates 10,000 noise datasets replicating the variance of the real data. Paired *t*‐tests on each simulation generated a null distribution of cluster sizes. Only voxels with *p* < 0.01 entered clustering, yielding cluster size thresholds for different significance levels (α). For each map, the minimum cluster size corresponding to α < 0.05 (with NN = 1) was computed, reducing the likelihood of false positives.

Post hoc power analyses were then conducted for ReHo‐shift and ALFF‐shift at rest1 and rest2. Significant voxel masks from the four paired *t*‐tests were used to compute average Z‐values, which were converted to effect sizes (Cohen's *d*) by dividing by √n (Cohen 2013). These values were entered into G*Power (v3.1.9.7) (Faul et al. 2009), using a one‐tailed alpha of 0.05.

#### 2.5.3 Conjunction Maps and Vertex‐Wise Correlations

To evaluate the robustness of our findings and compare ALFF‐shift and ReHo‐shift maps, four conjunction maps were generated: (1–2) ALFF‐shift‐rest1*rest2 and ReHo‐shift‐rest1*rest2 and (3–4) ALFF‐shift‐rest1*ReHo‐shift‐rest1 and ALFF‐shift‐rest2*ReHo‐shift‐rest2. Specifically, the conjunction maps were created by combining the significant result masks after applying the ClustSim threshold. The AFNI *whereami* tool was employed to identify the areas in each conjunction map. Since, during preprocessing the runs were registered in MNI space, the CA_ML_18_MNIA (Cox 1996; Eickhoff et al. 2005) atlas was used as the anatomical reference.

To further support the results obtained from the four conjunction maps, we also computed vertex‐wise correlations to assess the following: (a) ALFF test–retest reliability between rest1 and rest2 runs; (b) ReHo test–retest reliability between rest1 and rest2 runs; (c) the cross‐modal correlation between ALFF and ReHo values in rest1; and (d) the cross‐modal correlation between ALFF and ReHo values in rest2. For this purpose, the collection of features from *neuromaps* (Markello et al. 2022) was utilized. Initially, *neuromaps* was used to convert ALFF‐shift and ReHo‐shift maps to a common space of choice (*fsaverage*) (Thomas Yeo et al. 2011; Wu et al. 2018). Then, the vertex‐wise spatial correlation (Spearman rho) between a shift map and the other (i.e., ALFF‐shift‐rest1 vs. rest2 and ReHo‐shift‐rest1 vs. rest2) was computed (Markello et al. 2022). To estimate the statistical significance of the correlation values, null models were obtained by rotating the reference images to derive the null distribution of correlation coefficients (spatial nulls, *n* = 1000) (Vázquez‐Rodríguez et al. 2019). An analogous analysis was performed to obtain cross‐modal correlations between ALFF and ReHo values across both the rest1 and rest2 sessions. Finally, as a sensitivity analysis, we computed vertex‐wise correlations between ALFF‐shift and mALFF‐shift maps for both rest1 and rest2 runs, in order to verify the robustness of ALFF results to normalization procedures.

### 2.6 Secondary Analysis: Neurochemical and Topological Enrichment

Considering the existing literature on LSD (Canal and Murnane 2017; Halberstadt and Geyer 2011; Moreno et al. 2011; Passie et al. 2008; Perry and Perry 1995; Seeman et al. 2005) and the available receptor maps at the time of study conduction, the following spatial features were retrieved from *neuromaps*: acetylcholine receptors (both nicotinic—AChN—and muscarinic—AChM1), acetylcholine transporter (VAChT), dopamine receptors (D1 and D2), dopamine transporter (DAT), glutamate receptors (mGlutR5 and NMDA), serotonin receptors (5‐HT1A, 5‐HT1B, 5‐HT2A, and 5‐HT4) and transporter (SERT), opioid receptors (morphine—MOR—and kappa—KOR), cannabinoid receptors (CB1), and GABA receptors (GABAa).

As above, *neuromaps* was used to convert ALFF/ReHo‐shift maps to a common space of choice (*fsaverage*) (Buckner et al. 2011; Wu et al. 2018). *Neuromaps* was then used to estimate the vertex‐wise spatial correlation (Spearman rho) between the group contrast (now in *fsaverage*) and the selected feature map (the single receptor/transporter density map) across the whole surface (Markello et al. 2022). To estimate statistical significance, null models were computed by rotating the reference images and deriving the null distribution of correlation coefficients (spatial nulls, *n* = 1000) (Vázquez‐Rodríguez et al. 2019). *p*‐values were adjusted for multiple comparisons using false discovery rate (FDR‐*p*) correction (Benjamini and Hochberg 1995). As a sensitivity analysis, receptor‐enrichment procedures were repeated using mALFF‐shift maps.

## 3 Results

### 3.1 Descriptive Statistics

As reported in the original study (Carhart‐Harris et al. 2016), one subject did not complete the fMRI scans due to anxiety, while four other subjects were discarded from the group analyses due to excessive head movement (see Section e2 in the Supporting Information). The final sample included 15 subjects (four females; mean age, 30.5 ± 8.0 years).

### 3.2 LSD‐Induced Reconfigurations in ALFF and ReHo: Test–Retest Reliability in Rest1 and Rest2 Runs

**FIGURE 1:**
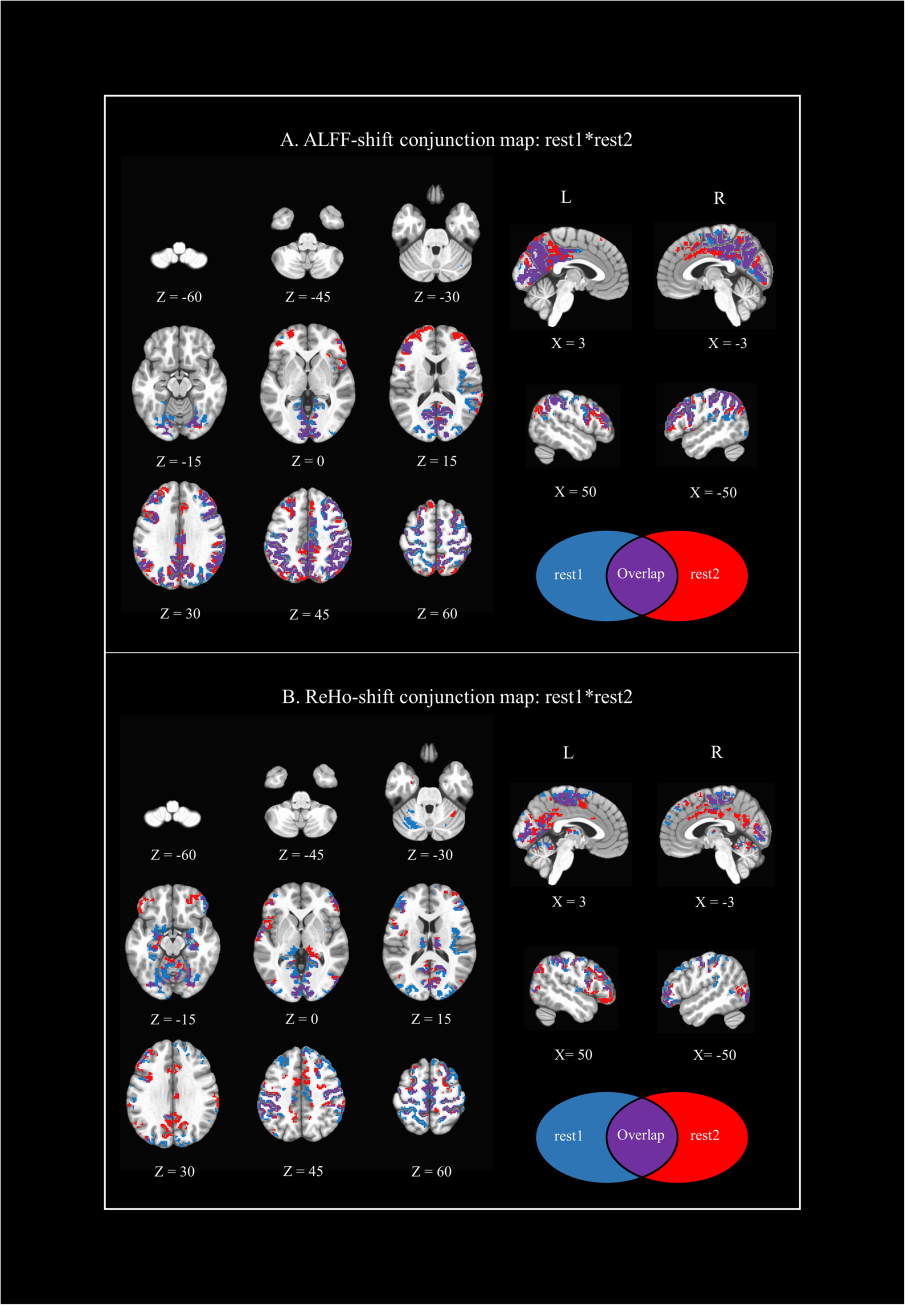
Conjunction maps between rest1*rest2 ALFF/ReHo LSD‐PBO *t*‐tests results (i.e., ALFF/ReHo‐shift). (1A) ALFF‐shift‐rest1*rest2; (1B) ReHo‐shift‐rest1*rest2. Rest1 and rest2 z maps for both ALFF and ReHo results were corrected for multiple comparisons (cluster‐based correction, *p* < 0.05) prior to the generation of the conjunction maps.

ALFF values demonstrated a statistically significant widespread decrease under LSD compared to PBO (i.e., a negative ALFF‐shift) in both rest1 and rest2 runs (Figure S1). Conversely, no ALFF increases were observed. The regions exhibiting negative ALFF‐shift values in both runs (Figure 1A) included the following: bilateral primary visual cortex, cuneus, precuneus, retrosplenial cortex, right fusiform gyrus, supramarginal gyrus, posterior cingulate, dorsolateral prefrontal cortex (dlPFC), middle frontal gyrus, inferior frontal gyrus (IFG), right supplementary motor area (SMA), sensorimotor cortex (SMC), and posterior parietal cortex (PPC). See Table 1A for region details and coordinates.

**TABLE 1:** Overlapping regions in conjunction maps between ALFF‐shift‐rest1*rest2 (1A) and ReHo‐shift‐rest1*rest2 (1B).

A. Conjunction map between ALFF‐shift‐rest1*rest2		
ROI (*n* voxels)	CM [x; y; z]	Anatomical regions (% on total ROI size)
ROI 1 (8913)	+9.6; +56.6; +30.4	Precuneus (R: 11.7%; L: 8.3%), L inferior parietal lobule (11.5%), calcarine gyrus (R: 7.9%; L: 9.7%), posterior cingulate cortex (R: 5.6%; L: 2.6%), lingual gyrus (R: 2.5%; L: 4.9%), cuneus (R: 4.3%; L: 3.3%), R SMA (3.7%), L middle occipital gyrus (3.0%), L angular gyrus (2.6%), L postcentral gyrus (2.6%), L superior parietal lobule (2.5%), L supramarginal gyrus (2.3%)
ROI 2 (3095)	−47.7; +46.3; +41.8	R inferior parietal lobule (22.6%), R angular gyrus (21.6%), R supramarginal gyrus (19.2%), R postcentral gyrus (17.5%), R superior temporal gyrus (4.3%), R middle occipital gyrus (4.2%), R superior parietal lobule (4.0%), R superior occipital gyrus (2.9%)
ROI 3 (1548)	−45.1; −26.5; +26.7	R middle frontal gyrus (50.8%), R inferior frontal gyrus (p. opercularis 21.3%; p. triangularis 18.0%), R precentral gyrus (7.2%), R Rolandic operculum (2.0%)
ROI 4 (1090)	−30.6; +3.4; +54.5	R precentral gyrus (34.7%), R superior frontal gyrus (27.3%), R middle frontal gyrus (21.3%), R postcentral gyrus (7.5%), R SMA (2.9%)
ROI 5 (1083)	+27.7; +1.0; +54.4	L middle frontal gyrus (32.3%), L precentral gyrus (30.8%), L superior frontal gyrus (17.5%), L postcentral gyrus (15.3%)
ROI 6 (730)	+42.0; −37.2; +21.2	L inferior frontal gyrus (p. triangularis) (52.0%), L middle frontal gyrus (47.2%)
ROI 7 (421)	+44.1; −8.7; +30.3	L precentral gyrus (50.1%), L inferior frontal gyrus (p. opercularis 30.2%; p. triangularis 4.7%), L middle frontal gyrus (13.7%)
ROI 8 (323)	−18.9; +74.1; −9.5	R lingual gyrus (62.7%), R fusiform gyrus (27.7%), R cerebellum (VI) (4.5%), R inferior occipital gyrus (3.6%)
ROI 9 (165)	+19.7; +87.5; +23.4	L middle occipital gyrus (45.1%), L superior occipital gyrus (42.8%), L cuneus (12.0%)
ROI 10 (63)	+7.4; +51.0; +44.2	L precuneus (99.7%)
ROIs < 50 voxels		R insula lobe

B. Conjunction map between ReHo‐shift‐rest1*rest2		
ROI (*n* voxels)	CM [x; y; z]	Anatomical regions (% on total ROI size)
ROI 1 (3013)	−2.9; +70.4; +2.3	Calcarine gyrus (R: 17.7%; L: 19.8%), lingual gyrus (R: 14.7%; L: 11.1%), fusiform gyrus (R: 7.2%; L: 3.3%), precuneus (R: 3.2%; L: 4.6%), cuneus (R: 2.3%; L: 3.4%), L inferior occipital gyrus (2.3%), R cerebellum (VI) (2.0%)
ROI 2 (568)	+48.9; +37.5; +44.5	L inferior parietal lobule (62.9%), L postcentral gyrus (10.1%), L supramarginal gyrus (7.3%)
ROI 3 (519)	+42.9; −41.0; +14.3	L inferior frontal gyrus (p. triangularis 55.6%), L middle frontal gyrus (42.0%)
ROI 4 (475)	−40.8; +17.2; +51.6	R precentral gyrus (62.7%), R postcentral gyrus (22.8%), R middle frontal gyrus (3.1%)
ROI 5 (450)	+6.6; +23.9; +58.8	L SMA (36.7%), L paracentral lobule (36.5%), L precuneus (18.7%), L posterior cingulate cortex (3.1%)
ROI 6 (419)	−35.2; +40.4; +58.0	R postcentral gyrus (57.0%), R superior parietal lobule (18.8%), R inferior parietal lobule (14.8%), R supramarginal gyrus (6.7%)
ROI 7 (395)	+39.2; +18.7; +52.7	L postcentral gyrus (54.4%), L precentral gyrus (40.3%)
ROI 8 (393)	−5.9; +17.8; +56.2	SMA (R: 60.1%; L: 3.7%), R posterior cingulate cortex (16.7%), R paracentral lobule (16.7%)
ROI 9 (387)	−47.5; −42.7; −0.4	R inferior frontal gyrus (p. orbitalis 31.1%; p. triangularis 30.5%), R middle frontal gyrus (24.7%), R middle orbital gyrus (12.4%)
ROI 10 (341)	−43.1; +79.2; +2.8	R middle occipital gyrus (49.6%), R inferior occipital gyrus (25.9%), R middle temporal gyrus (19.4%)
ROI 11 (317)	+22.4; +8.3; +64.7	L superior frontal gyrus (43.2%), L precentral gyrus (41.3%), L paracentral lobule (5.9%), L middle frontal gyrus (5.2%)
ROI 12 (292)	+50.5; −2.4; +15.8	L precentral gyrus (36.3%), L Rolandic operculum (20.8%), L inferior frontal gyrus (p. opercularis 19.0%), L superior temporal gyrus (17.3%), L temporal pole (4.8%)
ROI 13 (237)	+1.2; +16.7; +11.5	Thalamus (R: 43.8%; L: 50.5%)
ROI 14 (201)	−24.4; −13.6; +56.8	R superior frontal gyrus (67.7%), R middle frontal gyrus (24.9%), R SMA (3.2%)
ROI 15 (183)	−29.3; +22.7; −14.8	R hippocampus (61.7%), R parahippocampal gyrus (33.6%)
ROI 16 (177)	+26.1; +16.9; −15.0	L hippocampus (57.3%), L amygdala (22.6%), L parahippocampal gyrus (13.6%)
ROI 17 (112)	+43.2; +76.7; +36.4	L middle occipital gyrus (25.6%), L angular gyrus (21.5%)
ROI 18 (112)	−20.3; +11.1; +70.0	R superior frontal gyrus (69.2%), R SMA (16.3%), R precentral gyrus (12.9%)
ROI 19 (85)	+44.1; +79.0; +2.6	L middle occipital gyrus (88.8%)
ROIs < 50 voxels		L cerebellum (IV‐V), L cerebellum (VI), cerebellar Vermis (3), cerebellar vermis (4/5), R and L superior medial gyrus, R amygdala, L superior parietal lobule

Abbreviations: CM, center of mass; L, left; R, right; ROI, region of interest.

Similarly to ALFF, ReHo showed several regions with lower, but not higher, values in LSD compared to PBO conditions in both rest runs (see Figure S2). The regions exhibiting negative ReHo‐shift values in both rest1 and rest2 runs (Figure 1B) included the following: bilateral supramarginal gyrus, bilateral IFG, SMA, fusiform gyrus, primary visual cortex, SMC, and dlPFC (where significant ALFF reductions in LSD were also present), left superior temporal gyrus, bilateral hippocampus, bilateral thalamus, amygdala, and bilateral cerebellum (changes exclusive to ReHo results). See Table 1B for region details and coordinates. Results from mALFF‐shift analyses were highly consistent with those from ALFF, confirming widespread decreases under LSD. The only differences concerned minor variations in cluster extent and the emergence of some positive shifts, likely reflecting relative effects introduced by normalization (Figure S3).

Post hoc power analyses are available in Table S1. The achieved power for ALFF‐shift analyses was 81.1% at rest1 and 81.5% at rest2; for ReHo‐shift analyses, achieved power was 81.5% at rest1 and 79.6% at rest2.

Vertex‐wise correlations between rest1 and rest2 runs showed that test–retest reliability was high for ALFF‐shift (rho = 0.80, *p* = 0.001) and moderate for ReHo‐shift (rho = 0.46, *p* = 0.001). Vertex‐wise correlations between ALFF‐shift and mALFF‐shift maps, as a sensitivity analysis, showed strong correlations both for rest1 (*r* = 0.96, *p* < 0.001) and rest2 (*r* = 0.96, p < 0.001). See Figures S4 and S5 for a graphical representation of results on cortical surfaces.

### 3.3 LSD‐Induced Reconfigurations: Comparison Between ALFF and ReHo Values

**FIGURE 2:**
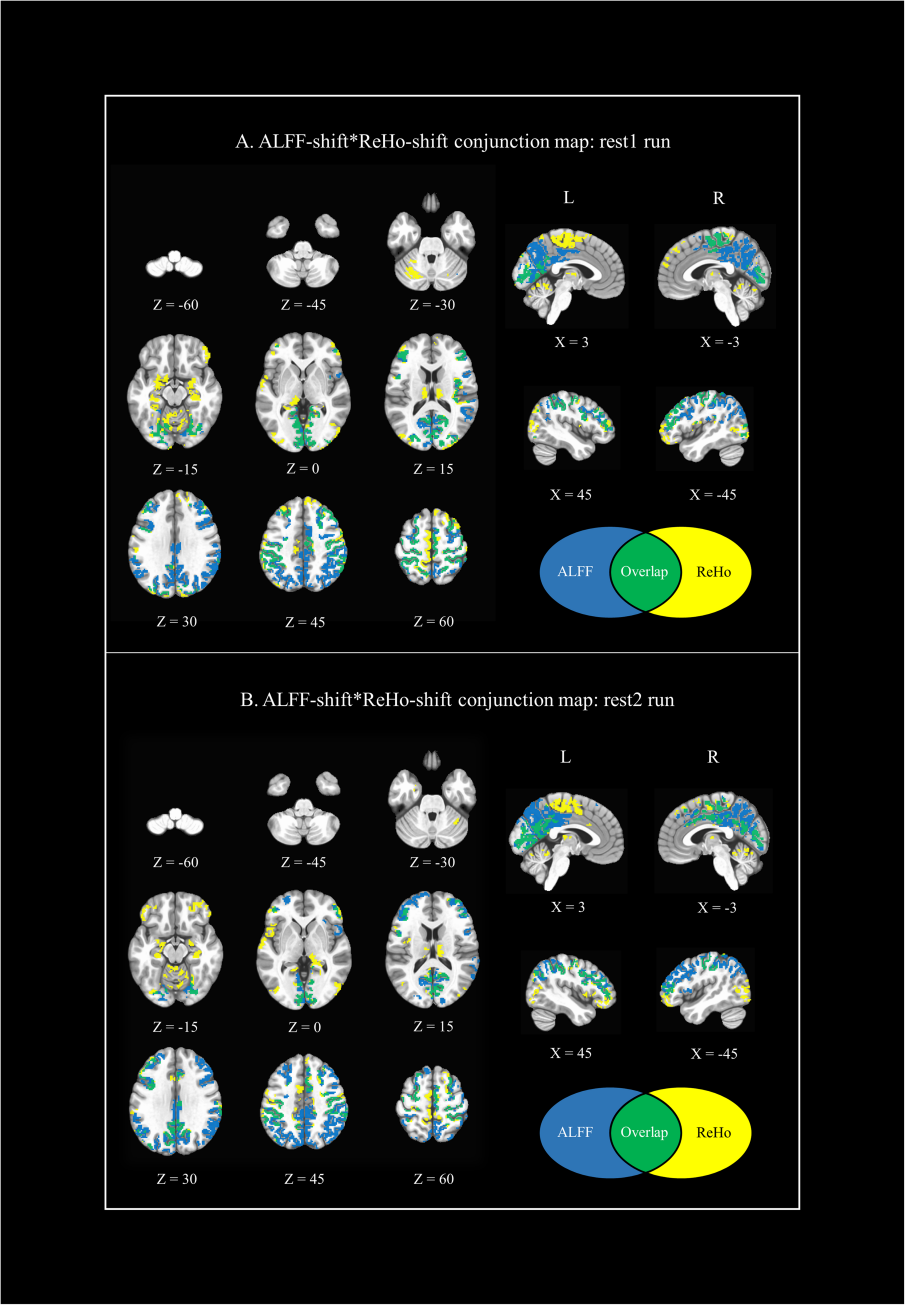
Conjunction maps between ALFF*ReHo LSD‐PBO *t*‐tests results (i.e., ALFF‐shift and ReHo‐shift) at rest1 and rest2. (2A) ALFF‐shift*ReHo‐shift‐rest1; (2B) ALFF‐shift*ReHo‐shift‐rest2. Rest1 and rest2 z maps for both ALFF and ReHo results were corrected for multiple comparisons (cluster‐based correction, *p* < 0.05) prior to the generation of the conjunction maps.

ALFF‐shift*ReHo‐shift conjunction maps (during rest1 and rest2) are visualized in Figure 2; overlapping regions are listed in Table 2. Overlapping regions between ALFF‐shift and ReHo‐shift were localized bilaterally in the occipital lobe, frontoparietal cortex, and sensorimotor regions. Overall, ALFF was more sensitive to LSD‐induced changes in dorsal brain regions, whereas ReHo highlighted LSD‐induced changes in more ventral brain regions and the cerebellum. ALFF and ReHo results were moderately correlated (rest1: rho = 0.36, *p* = 0.001; rest2: rho = 0.56, *p* = 0.001; see Figure S4).

**TABLE 2:** Overlapping regions in conjunction maps between ALFF‐shift*ReHo‐shift during rest1 (2A) and rest2 (2B).

A. Conjunction map between ALFF‐shift*ReHo‐shift‐rest1		
ROI (*n* voxels)	CM [x; y; z]	Anatomical regions (% on total ROI size)
ROI 1 (4113)	−0.9; +70.0; +1.4	Calcarine gyrus (R: 16.3%; L: 19.1%), lingual gyrus (R: 14.9%; L: 14.3%), fusiform gyrus (R: 6.4%; L: 3.9%), L cerebellum (VI) (4.8%), precuneus (R: 2.8%; L: 3.9%), cuneus (R: 2.9%; L: 2.1%), L inferior occipital gyrus (2.5%)
ROI 2 (2070)	+36.7; +29.2; +53.4	L inferior parietal lobule (27.1%), L postcentral gyrus (24.6%), L precentral gyrus (18.2%), L superior frontal gyrus (7.2%), L superior parietal lobule (5.4%), L supramarginal gyrus (3.2%), L precuneus (2.8%), L middle frontal gyrus (2.5%)
ROI 3 (1407)	−42.6; + 31.1; +52.6	R postcentral gyrus (33.3%), R precentral gyrus (23.8%), R inferior parietal lobule (16.5%), R superior parietal lobule (9.5%), R supramarginal gyrus (7.5%), R middle frontal gyrus (2.3%)
ROI 4 (910)	+38.2; −35.9; +27.0	L middle frontal gyrus (60.6%), L inferior frontal gyrus (p. triangularis) (31.2%), L superior frontal gyrus (6.9%)
ROI 5 (781)	−8.1; +15.5; +55.7	SMA (R: 44.6%; L: 2.0%), R posterior cingulate cortex (28.7%), R paracentral lobule (9.8%), R superior frontal gyrus (8.2%), R precentral gyrus (2.3%)
ROI 6 (720)	−42.3; −35.9; 27.3	R middle frontal gyrus (78.0%), R inferior frontal gyrus (p. triangularis) (16.7%), R superior frontal gyrus (2.8%)
ROI 7 (569)	−31.4; +86.3; +11.0	R middle occipital gyrus (44.4%), R inferior occipital gyrus (23.1%), R superior occipital gyrus (17.8%), R cuneus (9.5%)
ROI 8 (366)	−27.5; −14.7; +52.6	R middle frontal gyrus (52.3%), R superior frontal gyrus (42.3%)
ROI 9 (236)	+46.0; −4.7; +26.5	L precentral gyrus (65.7%), L inferior frontal gyrus (p. opercularis) (31.6%), L Rolandic operculum (2.6%)
ROI 10 (208)	+18.9; +88.8; +24.1	L superior occipital gyrus (47.1%), L middle occipital gyrus (37.8%), L cuneus (14.8%)
ROI 11 (189)	−39.7; +11.2; +16.1	R insula lobe (43.2%), R Rolandic operculum (20.1%), R supramarginal gyrus (12.2%), R Heschl's gyrus (5.2%), R inferior frontal gyrus (p. opercularis; 3.6%)
ROI 12 (159)	+35.8; +79.6; +32.7	L middle occipital gyrus (78.1%), L angular gyrus (4.0%)
ROI 13 (57)	−14.1; +93.4; +16.7	R cuneus (65.4%), R superior occipital gyrus (29.2%), R calcarine gyrus (5.4%)
ROIs < 50 voxels		R cerebellum (VI), R superior temporal gyrus

B. Conjunction map between ALFF‐shift*ReHo‐shift‐rest2		
ROI (*n* voxels)	CM [x; y; z]	Anatomical regions (% on total ROI size)
ROI 1 (3222)	−3.5; +67.8; +12.8	Calcarine gyrus (R: 17.7%; L: 18.9%), precuneus (R: 10.7%; L: 8.3%), lingual gyrus (R: 8.9%; L: 8.2%), cuneus (R: 3.1%; L: 8.0%), posterior cingulate cortex (R: 3.5%; L: 4.9%), R fusiform gyrus (2.9%)
ROI 2 (1137)	+43.3; −27.5; +21.3	L inferior frontal gyrus (p. triangularis 36.9%; p. opercularis 12.4%), L middle frontal gyrus (26.6%), L precentral gyrus (19.1%), L middle orbital gyrus (3.3%)
ROI 3 (731)	−46.6; +32.4; +47.9	R postcentral gyrus (49.6%), R supramarginal gyrus (32.4%), R inferior parietal lobule (9.5%), R superior parietal lobule (6.7%)
ROI 4 (699)	+47.6; +38.9; +44.6	L inferior parietal lobule (65.6%), L postcentral gyrus (11.8%), L supramarginal gyrus (7.6%)
ROI 5 (450)	−5.5; −4.2; +41.5	Posterior cingulate cortex (R: 56.2%; L: 4.9%), SMA (R: 23.6%; L: 2.9%), superior medial gyrus (R: 5.1%; L: 2.2%)
ROI 6 (403)	+23.3; +0.9; +59.5	L middle frontal gyrus (34.0%), L precentral gyrus (32.6%), L superior frontal Gyrus (26.9%), L paracentral lobule (3.6%)
ROI 7 (382)	−42.1; −46.1, +10.9	R middle frontal gyrus (60.7%), R inferior frontal gyrus (p. triangularis 32.9%; p. orbitalis 2.0%), R superior frontal gyrus (3.8%)
ROI 8 (367)	−37.2; +19.2; +51.8	R precentral gyrus (68.5%), R postcentral gyrus (17.7%)
ROI 9 (311)	−26.1; −14.8; +51.1	R superior frontal gyrus (50.8%), R middle frontal gyrus (38.3%), R precentral gyrus (4.9%)
ROI 10 (303)	−6.0; +19.8; +55.9	SMA (R: 59.9%; L: 3.2%), R paracentral lobule (16.3%), R posterior cingulate cortex (15.3%)
ROI 11 (281)	+46.4; +70.3; +35.4	L angular gyrus (52.2%), L middle occipital gyrus (15.5%), L middle temporal gyrus (2.5%)
ROI 12 (281)	+37.5; +19.8; +51.9	L postcentral gyrus (60.5%), L precentral gyrus (35.8%)
ROI 13 (131)	−22.9; +11.6; +67.3	R superior frontal gyrus (62.5%), R precentral gyrus (22.7%), R SMA (13.0%)
ROIs < 50 voxels		L fusiform gyrus, L inferior occipital gyrus

Abbreviations: CM, center of mass; L, left; R, right; ROI, region of interest.

### 3.4 Neurochemical Enrichment

**FIGURE 3:**
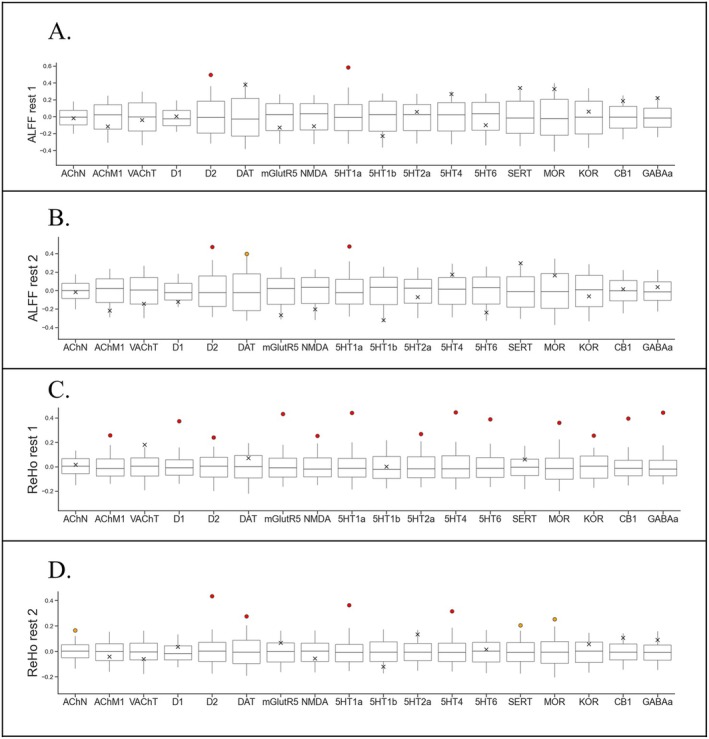
Neurochemical enrichment analysis. (3A) ALFF rest1; (3B) ALFF rest2; (3C) ReHo rest1; (3D) ReHo rest2. Boxes represent the interquartile range; whiskers represent 95% distributions of null coefficients. Values on the Y axis represent the observed correlation between the contrast of choice (ALFF and ReHo) and the selected receptor density map (Spearman rho). Red dots: FDR‐*p* < 0.05; orange dots: FDR‐*p* > 0.05 and uncorrected‐*p* < 0.05; crosses: uncorrected‐*p* > 0.05.

Overall, our enrichment analyses revealed only significant positive correlations between ALFF/ReHo‐shift and receptor maps. ALFF‐shift and ReHo‐shift maps showed a significant positive correlation with D2 and 5‐HT1A receptor maps (negative ALFF/ReHo‐shift corresponding to lower receptor density) across both rest1 and rest2 scans (Figure 3, ALFF‐shift rest1: D2 FDR‐*p* = 0.009, 5‐HT1A FDR‐*p* = 0.009; ALFF‐shift rest2: D2 FDR‐*p* = 0.045, 5‐HT1A FDR‐*p* = 0.036; ReHo‐shift rest1: D2 FDR‐*p* = 0.002, 5‐HT1A FDR‐*p* = 0.002; ReHo‐shift rest2: D2 FDR‐*p* = 0.006, 5‐HT1A FDR‐*p* = 0.006). 5‐HT1A and D2 cortical maps are available in Figure S6. Furthermore, LSD‐induced effects, as measured by ReHo‐shift, were spatially aligned across both scans with 5‐HT4 receptors (rest1: FDR‐*p* = 0.002; rest2: FDR‐*p* = 0.006).

ALFF results were relatively stable across scans, with an alignment with DAT approaching significance in rest2—although not surviving correction for multiple comparisons (Figure 3B; rest2: *p* = 0.005, FDR‐*p* = 0.258). The same analyses based on mALFF yielded consistent findings (Figure S7). In contrast, ReHo findings demonstrated significant alignment with various neurochemical receptors across both scans (D2: rest1 FDR‐*p* = 0.043, rest2 FDR‐*p* = 0.006; 5‐HT1A: rest1 FDR‐*p* = 0.002, rest2 FDR‐*p* = 0.006; 5‐HT4: rest1 FDR‐*p* = 0.002, rest2 FDR‐*p* = 0.006; MOR: rest1 FDR‐*p* = 0.004, but rest2 *p* = 0.024, FDR‐*p* = 0.086). However, ReHo‐shift during rest1 exhibited a spatial alignment with an even broader array of receptors in comparison to rest2 (AChM1: FDR‐*p* = 0.028; D1: FDR‐*p* = 0.002; mGluR5: FDR‐*p* = 0.002; NMDA: FDR‐*p* = 0.023; 5‐HT2A: FDR‐*p* = 0.023; 5‐HT6: FDR‐*p* = 0.002; KOR: FDR‐*p* = 0.002; CB1: FDR‐*p* = 0.002; GABAa: FDR‐*p* = 0.002) (Figure 3C,D).

## 4 Discussion

To the best of our knowledge, this study is the first to compute both ALFF and ReHo to investigate neural reconfigurations induced by psychedelics. We observed a reduction in ALFF and ReHo across multiple regions in the LSD compared to the PBO condition. These findings were consistent across both rest1 and rest2 sessions, showing good test–retest reliability. Notably, our neurochemical enrichment analysis demonstrated that LSD‐induced effects on ALFF and ReHo were specifically and consistently negatively aligned with D2 and 5‐HT1A receptors.

### 4.1 ALFF‐LSD‐Induced Reconfiguration Pattern

Our study reports a brain‐wide reduction in ALFF across both sensory and associative regions, including the somatosensory and visual cortices, as well as regions belonging to the default mode network (DMN) (precuneus and posterior cingulate) (Baghdadi et al. 2021) and the frontoparietal network (FPN) (dlPFC, inferior and posterior parietal lobule, and right temporoparietal junction) (Vendetti and Bunge 2014). The biological and functional significance of ALFF remains unclear (Merola et al. 2024). In fact, ALFF cannot be considered a metabolic proxy for brain activity, as it correlates with cerebral blood volume but not with the metabolic rate of glucose, the metabolic rate of oxygen, or cerebral blood flow (Deng et al. 2022). By definition, a decrease in ALFF corresponds to reduced power in the low‐frequency fMRI range (Yang et al. 2007; Zang et al. 2007), suggesting that local neuronal activity is dominated by desynchronized microstates rather than stable, strong fluctuations. This change may be driven by an increased contribution of higher‐frequency oscillations, which normally have lower amplitude and reflect more dynamic states (Klar et al. 2023; Thompson and Fransson 2015; Zhang et al. 2023). Consistently, LSD has been shown to induce increases in cross‐frequency co‐activation and high‐frequency harmonics, supporting a shift toward more diverse and flexible brain dynamics (Atasoy et al. 2017; Luppi et al. 2023).

From an information theory perspective, “noisy,” desynchronized patterns indicate higher entropy (Carhart‐Harris 2018; Carhart‐Harris et al. 2014). Thus, LSD‐induced ALFF reductions may reflect an increase in entropy, aligning with the EBH (Carhart‐Harris 2018; Carhart‐Harris et al. 2014). The EBH posits that typical waking consciousness operates near a critical zone, straddling order (low entropy) and disorder (high entropy). Studies have consistently reported LSD‐induced increases in brain entropy (Carhart‐Harris 2018; McCulloch et al. 2022), suggesting a shift closer to this critical zone, potentially enhancing information processing and explaining LSD‐related phenomena such as ego‐dissolution, sensory overload, and visual hallucinations (Atasoy et al. 2017; Lebedev et al. 2015; Luppi et al. 2023). Notably, Lebedev and colleagues reported an LSD‐induced increase in Sample Entropy across sensory and higher‐order networks (Lebedev et al. 2016), with a pattern that mirrors the ALFF reductions observed in our study. Future studies may investigate the relationship between ALFF and entropy to confirm their possible inverse association. In brief, reduced ALFF in LSD may reflect an amplification of high‐frequency harmonic activity, measurable as a relative reduction in the dominance of low‐frequency fluctuations. A smaller difference in power between low and high frequencies is a characteristic spectral feature of entropic increases (Damiani et al. 2019).

### 4.2 ReHo‐LSD‐Induced Reconfiguration Pattern

ReHo reductions were primarily observed within sensory areas such as the visual and sensorimotor cortices, as well as in subcortical regions including the thalamus and amygdala. Previous studies on the general population during cognitive tasks have documented that a brain‐wide reduction in ReHo is associated with increased between‐network connectivity and decreased within‐network connectivity (Damiani et al. 2022; Tommasin et al. 2018). Our results suggest the presence of similar dynamics under LSD. Previously, reductions in within‐network FC (referred to as “integrity”) and increases in between‐network FC (i.e., decreased “segregation” or “modularity”) were described during exposure to LSD—particularly in sensory regions—and were associated with visual hallucinations and ego‐dissolution (Bedford et al. 2023; Carhart‐Harris et al. 2016; Dai et al. 2023; Linguiti et al. 2023; Luppi et al. 2021; McCulloch et al. 2022; Moujaes et al. 2024; Müller et al. 2018; Tagliazucchi et al. 2016). Interestingly, LSD was found to exert differential effects on sensory versus associative areas; for instance, it increased brain‐wide connectivity in visual and sensorimotor cortices while reducing connectivity in associative areas (Preller et al. 2018). In line with these findings, Girn et al. (2022) observed a reduced differentiation between sensory unimodal and association multimodal areas, revealing a flattening of the brain's functional hierarchy under LSD. Consistent with these previous observations, we found a specific LSD‐induced reduction of ReHo in sensory regions, which is indicative of a fragmentation of network integrity.

Conversely, during propofol‐induced anesthesia, increases in ReHo have been observed (Huang et al. 2018) alongside heightened network integrity (Schrouff et al. 2011). In other words, states of “diminished consciousness” (such as anesthesia) display increased segregation, integrity, and ReHo, patterns essentially opposite to those observed in psychedelic states—which are often characterized as “expanded” states of consciousness (Carhart‐Harris 2018; Carhart‐Harris et al. 2014).

Finally, as for ALFF, ReHo reductions may also be consistent with an increase in entropy (Trevino et al. 2024). Previous investigations have consistently reported that higher ReHo values are associated with simpler forms of information processing (Jiang et al. 2015; Jiang and Zuo 2016), which may be reflected by lower levels of entropy according to EBH. In this regard, one study directly demonstrated a negative correlation between entropy and ReHo in a sample of patients with Alzheimer's disease (Wang et al. 2017).

### 4.3 Comparison Between ALFF and ReHo

ALFF‐ and ReHo‐shift patterns showed a moderate vertex‐wise correlation during both rest1 and rest2. Consistent with previous fMRI studies that employed both ALFF and ReHo (An et al. 2013; Shen et al. 2020; Sun et al. 2022; Wang et al. 2018; Yuan et al. 2013; Yue et al. 2020), these measures were found to provide both congruent and complementary information regarding neural functional dynamics. Concerning their similarities, ALFF and ReHo converged towards reductions in somatosensory and visual regions. This finding suggests a disruption of the brain functional hierarchy, favoring brain‐wide integration of the visual and somatosensory networks at the expense of segregation.

However, LSD‐induced reconfiguration also revealed notable differences between ALFF and ReHo. Specifically, ALFF selectively decreased in associative regions belonging to the DMN and FPN, whereas ReHo selectively decreased in ventral and subcortical regions such as the thalamus, amygdala, and cerebellum. Moreover, we observed that ALFF‐shift exhibited greater stability between rest1 and rest2 compared to ReHo, with vertex‐wise correlations of 0.80 versus 0.46, respectively. Previous studies have similarly reported that ALFF generally demonstrates slightly higher test–retest reliability than ReHo (Chen et al. 2017; Golestani et al. 2017). It should be noted, however, that test–retest stability of fMRI metrics varies across different measures and conditions. For example, general anesthesia—a state of diminished consciousness—has been shown to improve test–retest reliability of various fMRI metrics, including ALFF and ReHo (Vedaei et al. 2022). Therefore, it can be hypothesized that LSD, which is associated with expanded or altered consciousness, might reduce test–retest reliability by enhancing neural variability.

### 4.4 Neurochemical Enrichment

Neurochemical enrichment analyses confirmed that, although not replicated across both runs, the known main target of LSD, namely, 5‐HT2A, was significantly and spatially associated with ReHo results. Our hypothesis that LSD‐induced effects on ALFF and ReHo are spatially aligned with the receptor maps beyond 5‐HT2A alone was also confirmed, as D2 and 5‐HT1A receptors were consistently associated with both these measures across both runs. In this regard, it is important to note that previous studies have shown that LSD may have comparable or higher binding to 5‐HT1A (Ki ~ 3 nM) and D2 receptors (Ki ~ 2.5 nM) than to 5‐HT2A (Ki ~ 4 nM) (Ray 2010; Rickli et al. 2016). Notably, all the significant spatial correlations were positive, indicating that the greater the ALFF and ReHo reductions—i.e., the primary LSD‐induced effect we observed—the lower the receptor density. Furthermore, the overlap between ALFF‐/ReHo‐shift and the cortical distribution maps of 5‐HT1A and D2 receptors was minimal (see Figures 1, S4, and S6).

The full biological and functional significance of these results is difficult to posit, given the low sample size, the use of normative receptor profiles, and limitations imposed by receptor distributions (e.g., D2 receptors show low cortical distribution). Nevertheless, different hypotheses may be proposed. First, neurochemical enrichment analyses may reflect direct or indirect modulations rather than direct receptor binding. In fact, previous studies observed that FC reductions after MDMA intake were not linked to 5‐HT2A, as expected by direct binding potentials, but rather to 5‐HT1A (Dipasquale et al. 2019). Similarly, another study conducted on magnetoencephalography data rather than fMRI also showed no significant spatial association between LSD effects and 5‐HT2A receptors (Shinozuka et al. 2025). Secondly, a recent paper described that 5‐HT2A psychedelic agonism can alter neuronal and hemodynamic functioning (Padawer‐Curry et al. 2025). Current results may then be influenced by divergent neurovascular coupling, in line with ALFF reflecting more complex dynamics than simple neuronal activation/deactivation. Thirdly, neural populations rich in specific receptors such as 5‐HT1A and D2 may “prevent” LSD‐induced ALFF/ReHo reductions, in a similar manner to the “selective sparing” potential observed for similar receptors in other structural studies dealing with other neuropsychiatric disorders (Tarchi et al. 2025; Wiesman et al. 2024). Indeed, although LSD provokes silencing of serotonin receptors in the dorsal raphe through the activation of 5‐HT1A inhibitory autoreceptors (Aghajanian et al. 1968), 5‐HT1A agonists may induce burst firing in other brain regions, including those rich in noradrenergic and dopaminergic neurons (Díaz‐Mataix et al. 2005; Lejeune and Millan 1998; Piercey et al. 1994). In this regard, an early report showed that 5‐HT1A agonists may antagonize the effects of psychotomimetic drugs on dopaminergic neurons (Piercey et al. 1994), an observation that aligns with current results showing high 5‐HT1A receptor density as being associated with a lack of LSD‐induced ALFF/ReHo reductions.

Our results revealed that while ALFF remained relatively stable between rest1 and rest2, ReHo exhibited notable differences between these sessions. This suggests that ReHo may be more volatile and sensitive to short‐scale temporal dynamics under LSD, whereas ALFF might better reflect more stable characteristics of BOLD signals (see Section 4.3).

### 4.5 Limitations

A recent review by Linguiti et al. (2023) highlighted key challenges in the field of neuroimaging studies on psychedelics, including considerable variability in study designs, reliance on small and often overlapping samples, and inconsistent control of Type I error. To improve the reliability of our results, we repeated analyses on both rest1 and rest2 runs. All our results demonstrated moderate to robust test–retest reliability.

Despite the particular attention paid to addressing head motion during the preprocessing stages, a small yet significant difference in FD mean values still remained between LSD and PBO conditions (see Section e2 in the Supporting Information). Head motion can introduce artifacts in both ALFF and ReHo computations, potentially affecting our LSD‐PBO results. Nevertheless, the test–retest stability of our findings supports the robustness of the ALFF/ReHo results.

A potential limitation of this study is that the second resting‐state scan followed a music‐listening session, which may have influenced neural activity independently of LSD. Previous studies on this dataset have used both runs (McCulloch et al. 2022), a choice that is particularly valuable given the relatively small sample size. However, those studies typically averaged the two runs before analysis (Luppi et al. 2021; Speth et al. 2016), which may inadvertently mix potential music‐related effects with drug effects. In contrast, we analyzed the two runs separately and focused our interpretation on results consistently replicated across both scans, thereby reducing the likelihood that our main findings are driven by music‐related effects.

As discussed above, this is the first study to employ both ReHo and ALFF to investigate acute psychedelic‐induced neural dynamics. The lack of previous findings for direct comparison with our results limits our conclusions, so as to avoid overly speculative hypotheses. Despite this, we have sought to support our inferences by discussing our results in the context of various previous fMRI analysis methodologies and by incorporating neurochemical enrichment analyses. Future studies could strengthen these findings by directly comparing local fMRI measures with other noninvasive techniques, such as EEG or MEG, to better characterize the temporal and spatial aspects of psychedelic brain dynamics.

## 5 Conclusions

LSD‐induced reductions in ALFF and ReHo indicate a shift toward locally desynchronized brain function, aligning with previous findings of increased entropy and enhanced long‐distance connectivity. This reconfiguration of the brain's functional organization promotes greater integration of visual and somatosensory regions at the expense of their segregation. Neurochemical enrichment analyses further reveal that LSD‐induced alterations in these measures are reliably and negatively correlated with the density of several neurotransmitter systems, particularly 5‐HT1A and D2 receptors. These preliminary findings suggest that LSD may engage complex and dynamic neurochemical processes beyond its known 5‐HT2A receptor target, warranting further investigation.

## Author Contributions

**Paolo La‐Torraca‐Vittori:** conceptualization, data curation, methodology, validation, visualization, writing – original draft. **Livio Tarchi:** conceptualization, data curation, formal analysis, methodology, validation, visualization, writing – original draft. **Elisa Arrigo:** formal analysis, visualization. **Stefano Lanterna:** formal analysis, visualization. **Eleonora Tosi:** formal analysis, visualization. **Arne Doose:** methodology, software. **Fulvia Palesi:** writing – review and editing. **Doris Pischedda:** writing – review and editing. **Valdo Ricca:** supervision, writing – review and editing. **Paolo Fusar‐Poli:** supervision, writing – review and editing. **Stefano Damiani:** conceptualization, data curation, methodology, supervision, validation, visualization, writing – original draft, writing – review and editing.

## Funding

The authors received no specific funding for this work.

## Disclosure

The authors have nothing to report.

## Conflicts of Interest

The authors declare no conflicts of interest.

## Supporting information


**Figure S1:** Whole brain, voxel‐wise z maps displaying paired *t*‐tests between ALFF‐LSD and ALFF‐PBO (i.e., ALFF‐shift) during rest1 and rest2. A cluster‐based correction for multiple comparisons was applied to both z maps, ensuring that only clusters exceeding the minimum size threshold for statistical significance (α < 0.05; NN = 1, faces must touch) were retained. (e1A) Results for ALFF‐shift‐rest1 (minimum cluster size = 943); (e1B) Results for ALFF‐shift‐rest2 (minimum cluster size = 1630).
**Figure S2:** Whole brain, voxel‐wise z maps displaying paired *t*‐tests between ReHo‐LSD and ReHo‐PBO (i.e., ReHo‐shift) during rest1 and rest2. A cluster‐based correction for multiple comparisons was applied to both z maps, ensuring that only clusters exceeding the minimum size threshold for statistical significance (α < 0.05; NN = 1, faces must touch) were retained. (e2A) Results for ReHo‐shift‐rest1 (minimum cluster size = 281); (e2B) Results for ReHo‐shift‐rest2 (minimum cluster size = 295).
**Figure S3:** Whole brain, voxel‐wise z maps displaying paired *t*‐tests between mALFF‐LSD and mALFF‐PBO (i.e., mALFF‐shift) during rest1 and rest2. A cluster‐based correction for multiple comparisons was applied to both z maps, ensuring that only clusters exceeding the minimum size threshold for statistical significance (α < 0.05; NN = 1, faces must touch) were retained. (e3A) Results for mALFF‐shift‐rest1 (minimum cluster size = 652); (e3B) Results for mALFF‐shift‐rest2 (minimum cluster size = 547).
**Figure S4:** Whole brain, surface‐based z maps displaying paired *t*‐tests between ALFF/ReHo‐LSD and ALFF/ReHo‐PBO (i.e., ALFF/ReHo‐shift) during rest1 and rest2. (e4A) Results for ALFF‐shift‐rest1; (e4B) Results for ALFF‐shift‐rest2; (e4C) Results for ReHo‐shift‐rest1; (e4D) Results for ReHo‐shift‐rest2.
**Figure S5:** Whole brain, surface‐based z maps displaying paired *t*‐tests between mALFF‐LSD and mALFF‐PBO (i.e., mALFF‐shift) during rest1 and rest2. (e5A) Results for mALFF‐shift‐rest1; (e5B) Results for mALFF‐shift‐rest2.
**Figure S6:** Whole brain, surface‐based maps of receptors 5HT1A (e6A) and D2 (e6B).
**Figure S7:** Neurochemical enrichment analysis on mALFF‐shift. (e7A) mALFF‐shift rest1; (e7B) mALFF‐shift rest2. Boxes represent the interquartile range; whiskers represent 95% distributions of null coefficients. Values on the Y axis represent the observed correlation between mALFF and the selected receptor density map (Spearman rho). Red dots: FDR‐*p* < 0.05; orange dots: FDR‐*p* > 0.05 and uncorrected‐*p* < 0.05; crosses: uncorrected‐*p* > 0.05.
**Table S1:** Post hoc power analyses. Mean Z‐value refers to all significant voxels. Achieved power (1‐β error probability) was estimated based on Cohen's *d* values, considering an alpha error probability of 0.05 (two‐tailed) and a sample size of 15 subjects (14 degrees of freedom). The critical *t*‐value was 1.761.

## Data Availability

Original fMRI data was obtained from the OpenNeuro Dataset ds003059 (https://openneuro.org/datasets/ds003059/versions/1.0.0) (Carhart‐Harris et al. 2020). AFNI scripts used to analyze fMRI data, and the data that support the findings of this study are available from the corresponding author, PLTV, upon reasonable request and can be used for other works by including the first authors of this article as co‐authors of the work.
