# Supplementary material for: Knocking at the Doors of Perception: Relating LSD Effects on Low‐Frequency Fluctuations and Regional Homogeneity to Receptor Densities in fMRIf
Source: Eur J Neurosci. 2025 Nov 26;62(10):e70338. doi: 10.1111/ejn.70338 (PMC12658408; doi:10.1111/ejn.70338)
Supplement: Supplementary file 1 — Figure S1: Whole brain, voxel‐wise z maps displaying paired t‐tests between ALFF‐LSD and ALFF‐PBO (i.e., ALFF‐shift) during rest1 and rest2. A cluster‐based correction for multiple comparisons was applied to both z maps, ensuring that only clusters exceeding the minimum size threshold for statistical significance (α < 0.05; NN = 1, faces must touch) were retained. (e1A) Results for ALFF‐shift‐rest1 (minimum cluster size = 943); (e1B) Results for ALFF‐shift‐rest2 (minimum cluster size = 1630). Figure S2: Whole brain, voxel‐wise z maps displaying paired t‐tests between ReHo‐LSD and ReHo‐PBO (i.e., ReHo‐shift) during rest1 and rest2. A cluster‐based correction for multiple comparisons was applied to both z maps, ensuring that only clusters exceeding the minimum size threshold for statistical significance (α < 0.05; NN = 1, faces must touch) were retained. (e2A) Results for ReHo‐shift‐rest1 (minimum cluster size = 281); (e2B) Results for ReHo‐shift‐rest2 (minimum cluster size = 295). Figure S3: Whole brain, voxel‐wise z maps displaying paired t‐tests between mALFF‐LSD and mALFF‐PBO (i.e., mALFF‐shift) during rest1 and rest2. A cluster‐based correction for multiple comparisons was applied to both z maps, ensuring that only clusters exceeding the minimum size threshold for statistical significance (α < 0.05; NN = 1, faces must touch) were retained. (e3A) Results for mALFF‐shift‐rest1 (minimum cluster size = 652); (e3B) Results for mALFF‐shift‐rest2 (minimum cluster size = 547). Figure S4: Whole brain, surface‐based z maps displaying paired t‐tests between ALFF/ReHo‐LSD and ALFF/ReHo‐PBO (i.e., ALFF/ReHo‐shift) during rest1 and rest2. (e4A) Results for ALFF‐shift‐rest1; (e4B) Results for ALFF‐shift‐rest2; (e4C) Results for ReHo‐shift‐rest1; (e4D) Results for ReHo‐shift‐rest2. Figure S5: Whole brain, surface‐based z maps displaying paired t‐tests between mALFF‐LSD and mALFF‐PBO (i.e., mALFF‐shift) during rest1 and rest2. (e5A) Results for mALFF‐shift‐rest1; (e5B) Results fo [file EJN-62-0-s001.docx]

# Supporting Information

## e1. Anatomical scan and fMRI Data Acquisition

The screening process was conducted at Imperial’s Clinical Research Facility (ICRF) located on the

Hammersmith Hospital campus, while all study sessions took place at the Cardiff University Brain

Research Imaging Centre (CUBRIC).

Imaging was performed on a 3T GE HDx system. Structural scans were acquired using a 3D fast spoiled gradient echo scans in an axial orientation, with field of view = 256 × 256 × 192 mm and matrix size of 256 × 256 × 192, resulting in 1 mm isotropic voxel resolution. Other parameters were: TR/TE = 7.9/3.0 ms; inversion time = 450 ms; flip angle = 20°.

The fMRI scans were acquired using a gradient echo planar imaging sequence, TR/TE = 2000/35 ms, field of view = 220 mm, 64 × 64 acquisition matrix, parallel acceleration factor = 2, flip angle = 90°. Thirty-five oblique axial slices were acquired in an interleaved fashion, each 3.4 mm thick with zero slice gap (3.4 mm isotropic voxels). The precise length of each of the two fMRI scans was 7:20 minutes.

## e2. Pre-processing

Four complementary imaging software packages were used to preprocess the fMRI data: FMRIB Software Library (FSL) (1), AFNI (2), Freesurfer (3), and Advanced Normalization Tools (ANTS) (4). The following preprocessing steps were applied: 1) exclusion of the initial three volumes; 2) de-spiking (using *3dDespike* from AFNI); 3) slice timing correction (*3dTshift*, AFNI); 4) motion correction (*3dvolreg*, AFNI), in which each volume was aligned—using an in-house script—to the volume most representative of the entire dataset based on a least-squares criterion; 5) brain extraction (via BET, FSL); 6) rigid body registration to anatomical scans (twelve participants were registered using FSL’s BBR, one with Freesurfer’s bbregister, and two manually); 7) non-linear registration to the 2 mm MNI brain space (*SyN*, ANTS); 8) scrubbing (5), with a frame displacement (FD) threshold of 0.4 (the average proportion of scrubbed volumes was 0.4 ± 0.8% for PBO and 1.7 ± 2.3% for LSD, with a maximum of 7.1% of volumes scrubbed per scan); scrubbed volumes were replaced with the mean of the surrounding volumes. Additional steps included: 9) spatial smoothing with a full width at half maximum (FWHM) of 6 mm (*3dBlurInMask*, AFNI); 10) band-pass filtering between 0.01 and 0.08 Hz (*3dFourier*, AFNI); 11) linear and quadratic detrending (*3dDetrend*, AFNI); and 12) regression of nine nuisance regressors (all of which were band-pass filtered as in step 10). These nuisance regressors included six motion-related parameters (three translations, three rotations) and three anatomically related signals (not smoothed), which were: i) ventricles (from Freesurfer, eroded in 2 mm space); ii) draining veins (DV) (FSL’s CSF mask minus Freesurfer ventricles, eroded in 1 mm); and iii) local white matter (WM) (FSL’s WM mask minus Freesurfer subcortical grey matter, eroded in 2 mm). For local WM regression, the mean local WM time series for each voxel was computed using a 25 mm radius sphere centred on the voxel with AFNI’s *3dLocalstat* (6).

Resting-state functional connectivity analysis is highly sensitive to head motion (7); thus, particular attention was given to addressing motion during the pre-processing stages. Motion was primarily assessed using FD (7). Participants with more than 15% of volumes scrubbed when using an FD threshold of 0.5 were excluded. After excluding the four subjects for excessive motion, the scrubbing threshold was lowered to FD = 0.4. Among the four excluded subjects, the between-condition difference in mean FD was 0.323 ± 0.254; in contrast the mean FD difference in the final sample was 0.046 ± 0.032. Nonetheless, a significant difference in motion between conditions persisted among the included subjects (mean FD for PBO = 0.074 ± 0.032; mean FD for LSD = 0.12 ± 0.05, p = 0.0002). Further details about motion correction can be found in Section e3.

## e3. Motion correction during pre-processing

De-spiking has been found to enhance motion correction and yield more precise FD measurements (8). Employing a low-pass filter at 0.08 Hz effectively removes high-frequency motion artifacts (9). Six motion regressors were included as covariates in the linear regression model. It was decided not to use more than six regressors (such as the Friston 24-parameter model; (10)) because it could be redundant and might suppress genuine neural signals (11), especially given that other rigorous processes like scrubbing (5) and local white matter regression (6) were also applied. Draining veins and local white matter signals were incorporated into the pre-processing pipeline as anatomical regressors for reducing noise. Moreover, local white matter regression has been suggested to be more effective than global white matter regression in noise reduction (8).

It has previously been shown that head motion biases functional connectivity results in a distance-dependant manner (7). Therefore, as a quality control step, at the end of the preprocessing procedure, cloud plots were constructed to test for relationships between internode Euclidian distance and correlations between FD and RSFC across subjects. In cases in which motion is affecting the results, proximal nodes will have high FD-RSFC correlations and distal nodes will have low FD-RSFC correlations. This would result in a negative correlation between distance and FD-RSFC correlation. In the present dataset, the distance to FD-RSFC correlation was reported to be very close to zero for both the placebo and LSD conditions, suggesting that the extensive pre-processing measures had successfully controlled for distance-related motion artifacts.

## Figure S1

Whole brain, voxel-wise z maps displaying paired t-tests between ALFF-LSD and ALFF-PBO (i.e., ALFF-shift) during rest1 and rest2. A cluster-based correction for multiple comparisons was applied to both z-maps, ensuring that only clusters exceeding the minimum size threshold for statistical significance (α < 0.05; NN = 1, faces must touch) were retained. e1A) Results for ALFF-shift-rest1 (minimum cluster size = 943); e1B) Results for ALFF-shift-rest2 (minimum cluster size = 1630)


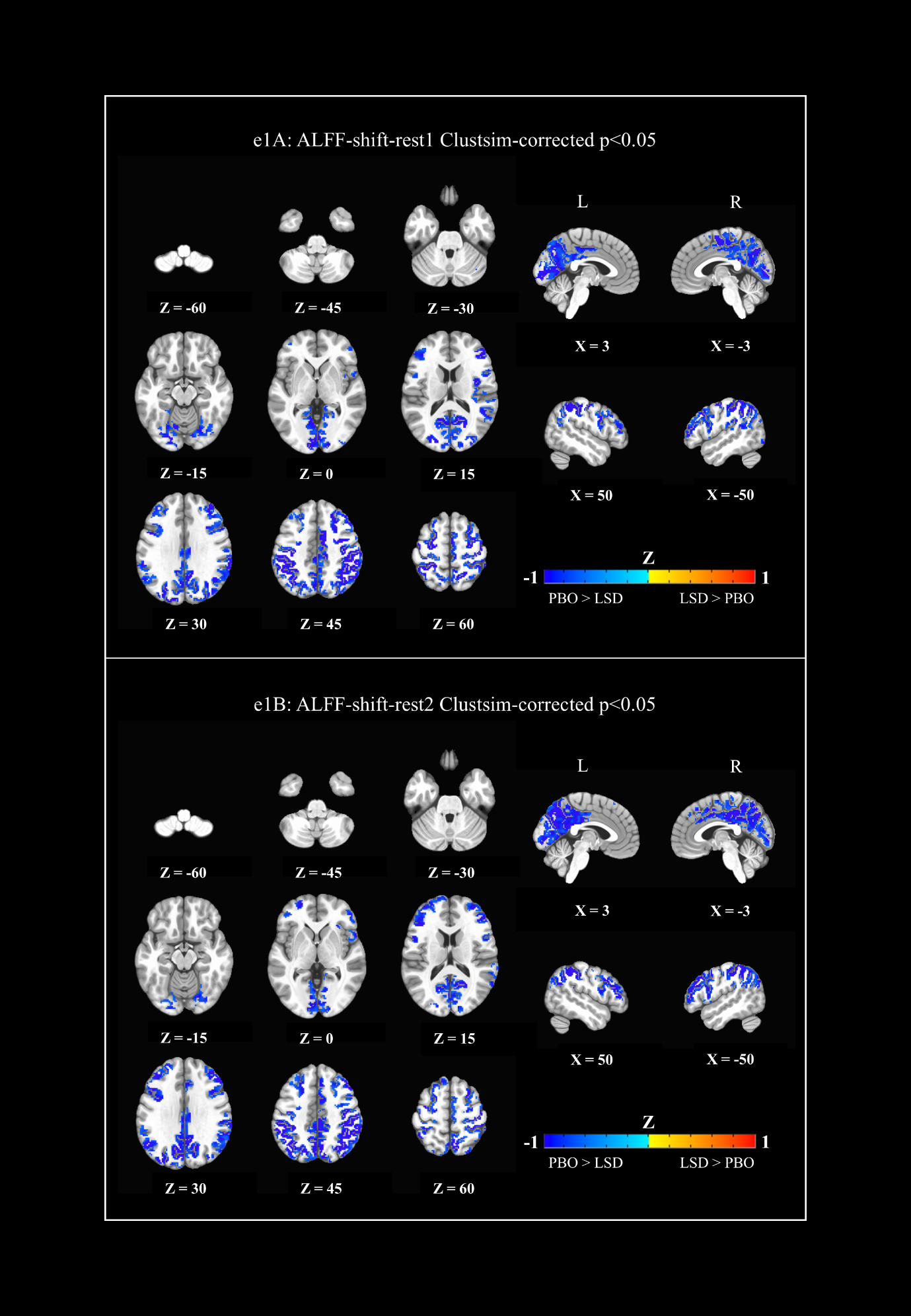


## Figure S2

Whole brain, voxel-wise z maps displaying paired t-tests between ReHo-LSD and ReHo-PBO (i.e., ReHo-shift) during rest1 and rest2. A cluster-based correction for multiple comparisons was applied to both z-maps, ensuring that only clusters exceeding the minimum size threshold for statistical significance (α < 0.05; NN = 1, faces must touch) were retained. E2A) Results for ReHo-shift-rest1 (minimum cluster size = 281); e2B) Results for ReHo-shift-rest2 (minimum cluster size = 295).


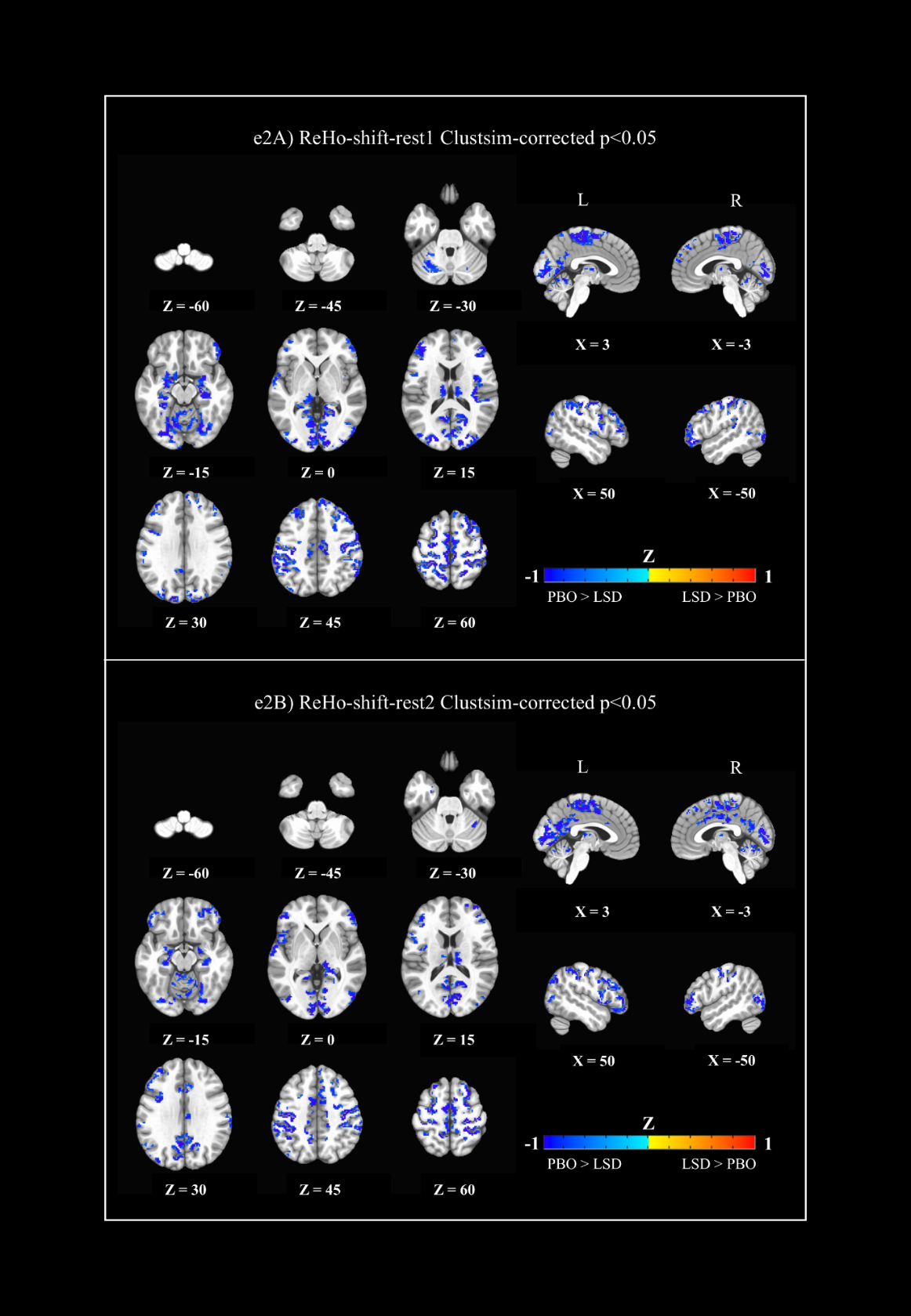


## Figure S3

Whole brain, voxel-wise z maps displaying paired t-tests between mALFF-LSD and mALFF-PBO (i.e., mALFF-shift) during rest1 and rest2. A cluster-based correction for multiple comparisons was applied to both z-maps, ensuring that only clusters exceeding the minimum size threshold for statistical significance (α < 0.05; NN = 1, faces must touch) were retained. e3A) Results for mALFF-shift-rest1 (minimum cluster size = 652); e3B) Results for mALFF-shift-rest2 (minimum cluster size = 547).


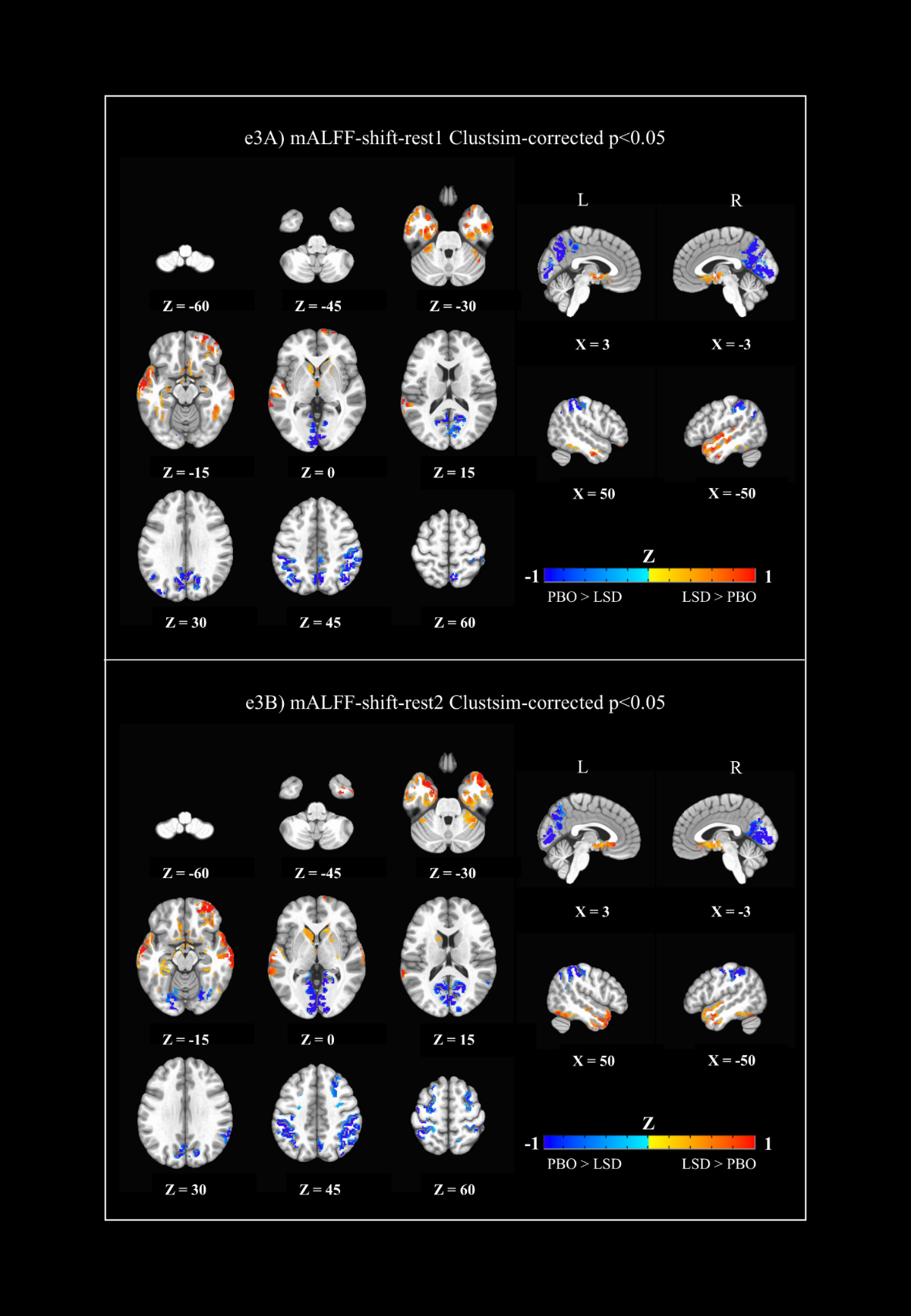


## Figure S4

Whole brain, surface-based z maps displaying paired t-tests between ALFF/ReHo-LSD and ALFF/ReHo-PBO (i.e., ALFF/ReHo-shift) during rest1 and rest2. e4A) Results for ALFF-shift-rest1; e4B) Results for ALFF-shift-rest2; e4C) Results for ReHo-shift-rest1; e4D) Results for ReHo-shift-rest2


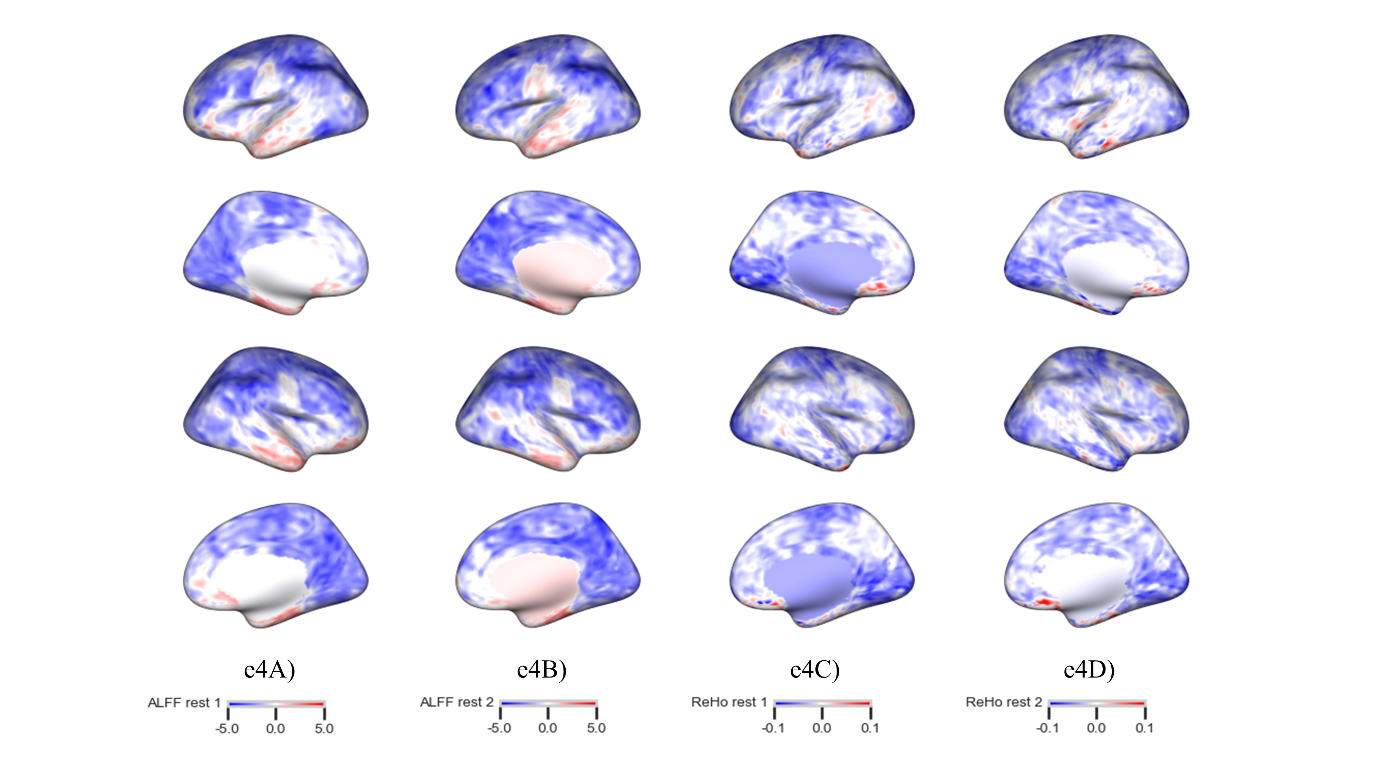


## Figure S5

Whole brain, surface-based z maps displaying paired t-tests between mALFF-LSD and mALFF-PBO (i.e., mALFF-shift) during rest1 and rest2. e5A) Results for mALFF-shift-rest1; e5B) Results for mALFF-shift-rest2.


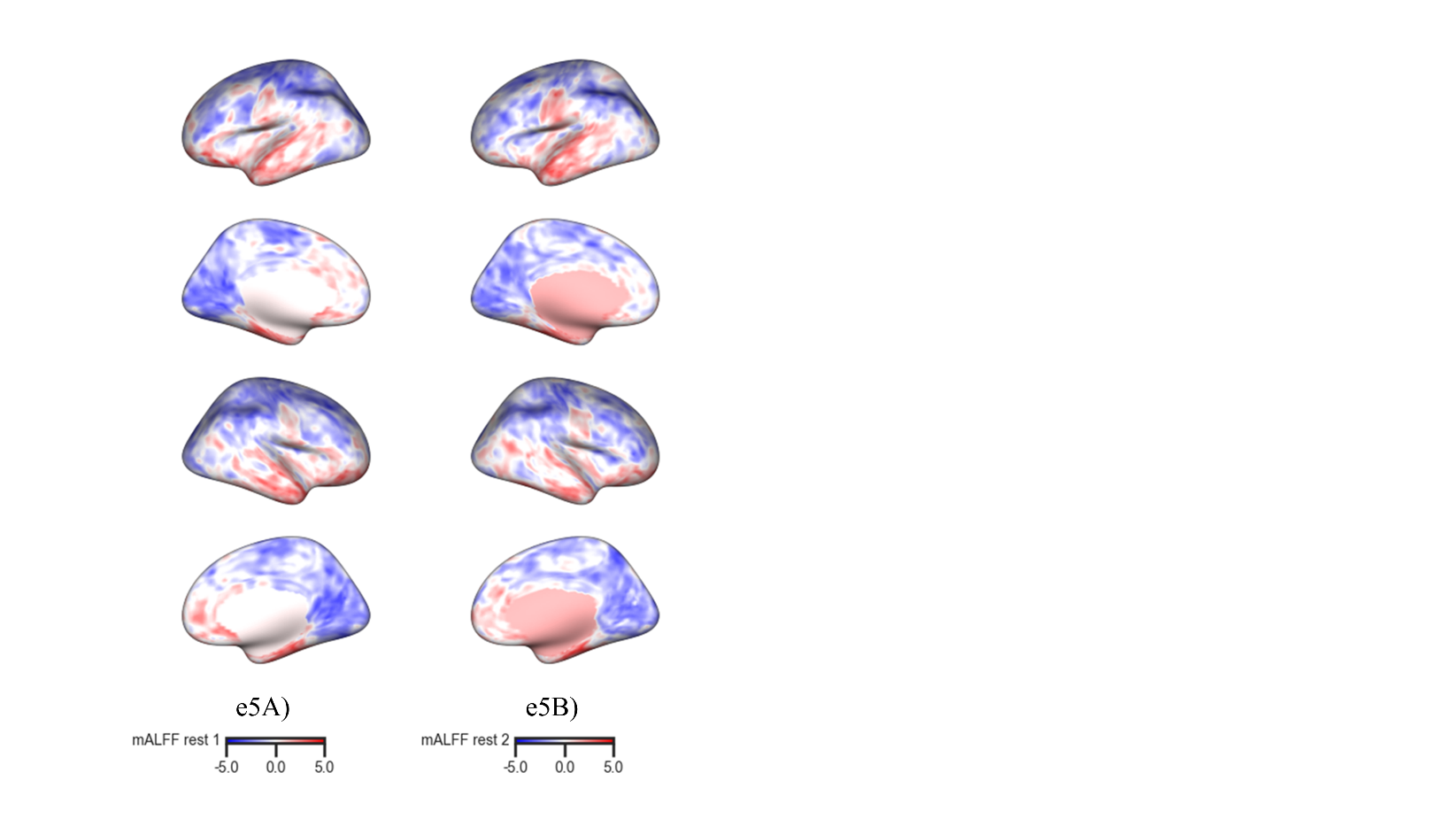


## Figure S6

Whole brain, surface-based maps of receptors 5-HT1A (e6A) and D2 (e6B)


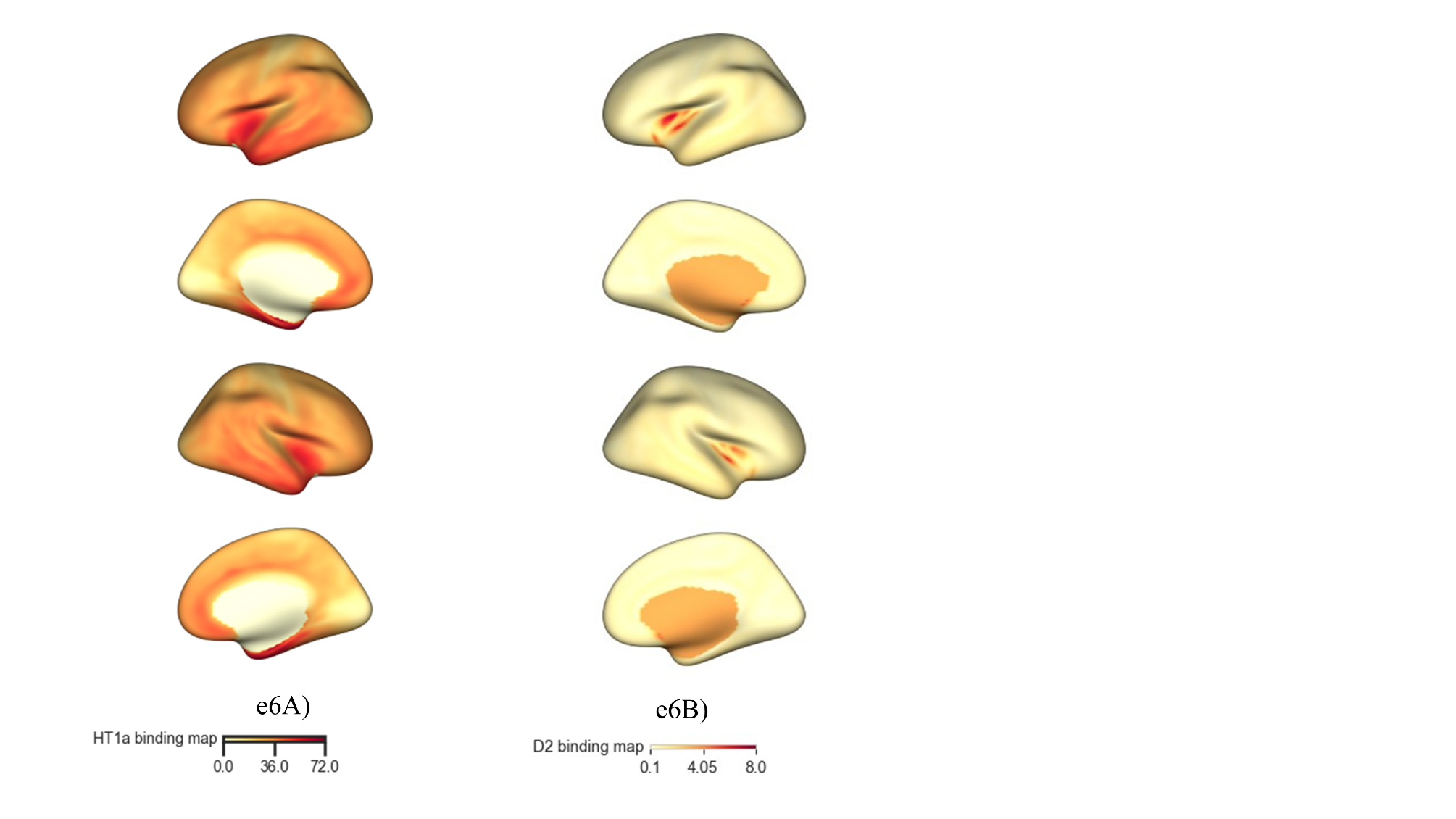


## Figure S7

Neurochemical enrichment analysis on mALFF-shift. e7A) mALFF-shift rest1; e7B) mALFF-shift rest2; Boxes represent the interquartile range; whiskers represent 95% distributions of null coefficients. Values on the Y axis represent the observed correlation between mALFF and the selected receptor density map (Spearman rho). Red dots: FDR-p < 0.05; Orange dots: FDR-p > 0.05 and uncorrected-p < 0.05; Crosses: uncorrected-p > 0.05.


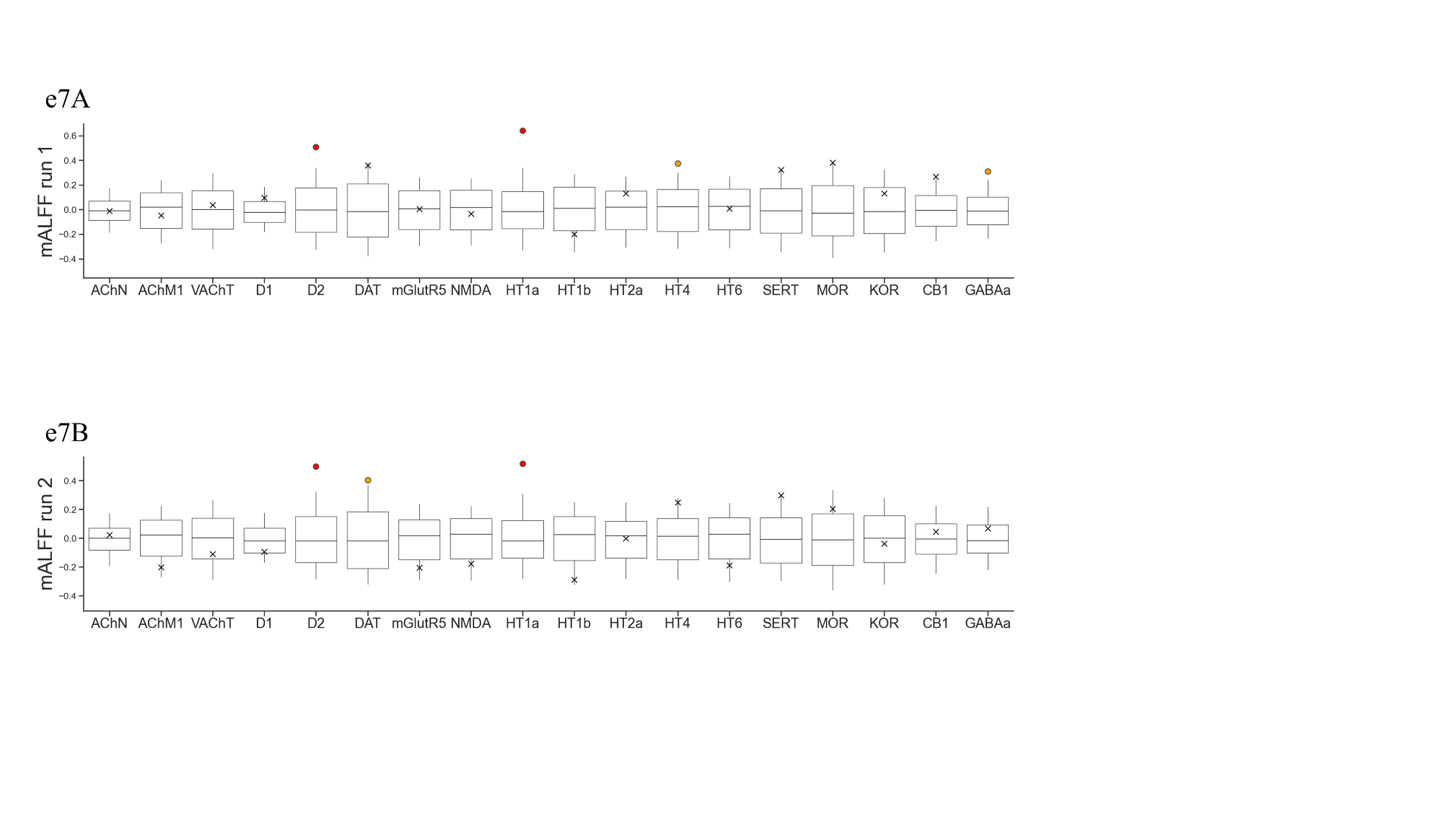


## Table S1

Post-hoc power analyses. Mean Z-value refers to all significant voxels. Achieved power (1- β error probability) was estimated based on Cohen’s d values, considering an alpha error probability of 0.05 (two-tailed) and a sample size of 15 subjects (14 degrees of freedom). The critical t-value was 1.761.

| Paired t-test  LSD-PBO | | Mean Z-value | Effect size (Cohen’s d) | Noncentrality parameter δ | Power (1-β) |
| --- | --- | --- | --- | --- | --- |
| ALFF-shift-rest1 | 2.658 | | 0.686 | 2.657 | 0.811 |
| ALFF-shift-rest2 | 2.673 | | 0.690 | 2.672 | 0.815 |
| ReHo-shift-rest1 | 2.674 | | 0.690 | 2.672 | 0.815 |
| Reho-shift-rest2 | 2.603 | | 0.672 | 2.602 | 0.796 |

## REFERENCES

1. Smith SM, Jenkinson M, Woolrich MW, Beckmann CF, Behrens TEJ, Johansen-Berg H, *et al.* (2004): Advances in functional and structural MR image analysis and implementation as FSL. *NeuroImage* 23: S208–S219.

2. Cox RW (1996): AFNI: software for analysis and visualization of functional magnetic resonance neuroimages. *Comput Biomed Res Int J* 29: 162–173.

3. Dale AM, Fischl B, Sereno MI (1999): Cortical Surface-Based Analysis. *NeuroImage* 9: 179–194.

4. Avants BB, Tustison N, Johnson H (2009): Advanced Normalization Tools (ANTS). *Insight j* 2: 1–35.

5. Power JD, Barnes KA, Snyder AZ, Schlaggar BL, Petersen SE (2012): Spurious but systematic correlations in functional connectivity MRI networks arise from subject motion. *NeuroImage* 59: 2142–2154.

6. Jo HJ, Saad ZS, Simmons WK, Milbury LA, Cox RW (2010): Mapping sources of correlation in resting state FMRI, with artifact detection and removal. *NeuroImage* 52: 571–582.

7. Power JD, Mitra A, Laumann TO, Snyder AZ, Schlaggar BL, Petersen SE (2014): Methods to detect, characterize, and remove motion artifact in resting state fMRI. *NeuroImage* 84: 320–341.

8. Jo HJ, Gotts SJ, Reynolds RC, Bandettini PA, Martin A, Cox RW, Saad ZS (2013): Effective Preprocessing Procedures Virtually Eliminate Distance-Dependent Motion Artifacts in Resting State FMRI. *J Appl Math* 2013: 1–9.

9. Satterthwaite TD, Elliott MA, Gerraty RT, Ruparel K, Loughead J, Calkins ME, *et al.* (2013): An improved framework for confound regression and filtering for control of motion artifact in the preprocessing of resting-state functional connectivity data. *NeuroImage* 64: 240–256.

10. Friston KJ, Williams S, Howard R, Frackowiak RSJ, Turner R (1996): Movement‐Related effects in fMRI time‐series. *Magn Reson Med* 35: 346–355.

11. Bright MG, Murphy K (2015): Is fMRI “noise” really noise? Resting state nuisance regressors remove variance with network structure. *NeuroImage* 114: 158–169.
